# The cAMP effector PKA mediates Moody GPCR signaling in *Drosophila* blood–brain barrier formation and maturation

**DOI:** 10.7554/eLife.68275

**Published:** 2021-08-12

**Authors:** Xiaoling Li, Richard Fetter, Tina Schwabe, Christophe Jung, Liren Liu, Hermann Steller, Ulrike Gaul

**Affiliations:** 1 Tianjin Cancer Hospital Airport Hospital, Tianjin Medical University Cancer Institute & Hospital Tianjin China; 2 Department of Biochemistry, Gene Center, Center of Integrated Protein Science (CIPSM), University of Munich Munich Germany; 3 Rockefeller University New York United States; 4 Janelia Farm Research Campus, Howard Hughes Medical Institute Ashburn United States; 5 Department of Gastrointestinal Cancer Biology, Tianjin Medical University Cancer Institute & Hospital; National Clinical Research Center for Cancer; Key Laboratory of Cancer Prevention and Therapy; Tianjin’s Clinical Research Center for Cancer Tianjin China; Howard Hughes Medical Institute, University of Oregon United States; University of California, Los Angeles United States

**Keywords:** blood-brain barrier, GPCR signaling, PKA, apical-basal polarity, septate junction, epithelium development, *D. melanogaster*

## Abstract

The blood–brain barrier (BBB) of *Drosophila* comprises a thin epithelial layer of subperineural glia (SPG), which ensheath the nerve cord and insulate it against the potassium-rich hemolymph by forming intercellular septate junctions (SJs). Previously, we identified a novel Gi/Go protein-coupled receptor (GPCR), Moody, as a key factor in BBB formation at the embryonic stage. However, the molecular and cellular mechanisms of Moody signaling in BBB formation and maturation remain unclear. Here, we identify cAMP-dependent protein kinase A (PKA) as a crucial antagonistic Moody effector that is required for the formation, as well as for the continued SPG growth and BBB maintenance in the larva and adult stage. We show that PKA is enriched at the basal side of the SPG cell and that this polarized activity of the Moody/PKA pathway finely tunes the enormous cell growth and BBB integrity. Moody/PKA signaling precisely regulates the actomyosin contractility, vesicle trafficking, and the proper SJ organization in a highly coordinated spatiotemporal manner. These effects are mediated in part by PKA’s molecular targets MLCK and Rho1. Moreover, 3D reconstruction of SJ ultrastructure demonstrates that the continuity of individual SJ segments, and not their total length, is crucial for generating a proper paracellular seal. Based on these findings, we propose that polarized Moody/PKA signaling plays a central role in controlling the cell growth and maintaining BBB integrity during the continuous morphogenesis of the SPG secondary epithelium, which is critical to maintain tissue size and brain homeostasis during organogenesis.

## Introduction

The blood–brain barrier (BBB) is a complex physical barrier between the nervous system and the peripheral circulatory system that regulates *central nervous system (CNS)* homeostasis to ensure proper neuronal function. The *Drosophila* BBB is established by a thin epithelium of subperineural glia (SPG), which ensheath and insulate the nervous system against the potassium-rich hemolymph by forming intercellular septate junctions (SJs) (Bainton et al., 2005; Carlson et al., 2000; Edwards et al., 1993). The SPG epithelium is formed as a result of a mesenchymal–epithelial transition (MET), similar to other secondary epithelia such as heart and midgut. SPG cells only increase in number in embryogenesis but not in larval development, and rather increase their size by polyploidization (Unhavaithaya and Orr-Weaver, 2012). Polyploidy in SPG is necessary to coordinate cell growth and BBB integrity either by Notch signaling or miR-285–Yki/Mask signaling during CNS development at the larval stage (Li et al., 2017; Unhavaithaya and Orr-Weaver, 2012; Von Stetina et al., 2018). SPG cells lack the apical markers present in primary epithelia (Crumbs, Bazooka), they have no contiguous zonula adherens and therefore rely on their SJ belt for epithelial cohesion, preventing paracellular diffusion and sealing the BBB (Schwabe et al., 2005; Stork et al., 2008; Tepass et al., 2001).

SJs are the crucial barrier junctions in invertebrates and functionally equivalent to vertebrate tight junctions (TJs); both junctions share claudins as key components (Izumi and Furuse, 2014). Structurally and molecularly, SJs are homologous to the vertebrate paranodal junction that forms between axons and myelinating glial cells adjacent to the node of Ranvier (for review, see Banerjee et al., 2006; Salzer, 2003; Salzer, 2015; Salzer et al., 2008). They consist of a core mutual interdependence protein complex, including transmembrane and cytoplasmic proteins, such as Neurexin-IV (Nrx-IV), Neuroglian (Nrg), the Na/K-ATPase (ATPα and Nrv2), the claudin Megatrachea (Mega), Sinous, Coracle (Cora), and the tetraspan Pasiflora protein family (Oshima and Fehon, 2011). In addition to the above-listed proteins, several GPI-anchored proteins, including Ly6-domain proteins Boudin, Crooked, Crimpled, and Coiled, Lachesin, Contactin, the tetraspan Pasiflora protein family, and Undicht, which are all found to be required for the SJ complex formation and proper membrane trafficking (Deligiannaki et al., 2015; Faivre-Sarrailh et al., 2004; Hijazi et al., 2011; Hijazi et al., 2009; Llimargas et al., 2004; Petri et al., 2019; Tempesta et al., 2017). The intracellular signaling pathways that control the assembly and maintenance of SJs are just beginning to be elucidated.

We have previously identified a novel GPCR signaling pathway that is required for the proper organization of SJ belts between neighboring SPG at the embryonic stage, consisting of the receptor Moody, two hetero-trimeric G proteins (Gαiβγ, Gαoβγ), and the RGS protein Loco. Both gain and loss of Moody signaling lead to non-synchronized growth of SPG cells, resulting in disorganized cell contacts and shortened SJs and, therefore, a leaky BBB (Schwabe et al., 2005; Schwabe et al., 2017). The phenotype of Moody is weaker than that of downstream pathway components including Loco and Gβ13F, suggesting that additional receptors provide input into the trimeric G protein signaling pathway. Gγ1 signaling has been shown to regulate the proper localization of SJ proteins in the embryonic heart (Yi et al., 2008). Despite its critical role in BBB formation, the underlying mechanisms connecting G protein signaling to continued SPG cell growth and the proper SJ organization during the development and maturation of BBB are still poorly understood.

One of the principal trimeric G protein effectors is adenylate cyclase (AC). AC is inhibited by the G proteins Gαi/Gαo and Gβγ, leading to decreased levels of the second messenger cAMP. The prime effector of cAMP, in turn, is cAMP-dependent protein kinase A (PKA), a serine/threonine kinase. PKA is inactive as a tetrameric holoenzyme, which consists of two identical catalytic and two regulatory subunits. Binding of cAMP to the regulatory units releases and activates the catalytic subunits (Taylor et al., 1990). PKA transmits the signal to downstream effectors by phosphorylating multiple substrates that participate in many different processes, from signal transduction to regulation of cell shape and ion channel conductivity (Shabb, 2001). In *Drosophila*, PKA has been studied as a component of GPCR signaling in the Hedgehog pathway during development (Li et al., 1995; Marks and Kalderon, 2011), and in neurotransmitter receptor pathways during learning and memory (Chen and Ganetzky, 2012; Guan et al., 2011; Li et al., 1996; Renger et al., 2000). PKA also regulates microtubule organization and mRNA localization during oogenesis (Lane and Kalderon, 1993; Lane and Kalderon, 1994; Lane and Kalderon, 1995). In vertebrates, cAMP/PKA signaling is known to play a central role within different subcellular regions, including the regulation of actomyosin contractility and localized cell protrusion in directional cell migration (Howe, 2004; Lim et al., 2008; Tkachenko et al., 2011); intracellular membrane trafficking (exocytosis, endocytosis, and transcytosis) in relation to the dynamics of epithelial surface domains in developmental processes and organ function (Wojtal et al., 2008). Moreover, cAMP/PKA signaling regulates endothelial TJ with diverse actions and unclear mechanisms in different endothelial cells models (Cong and Kong, 2020).

Here, we report results from a comprehensive in vivo analysis of the molecular and cellular mechanisms of Moody signaling in the SPG. We show that PKA is a key downstream effector responsible for the salient phenotypic outcomes, and that it acts by modulating actomyosin contractility via MLCK and Rho1. The strong phenotypic effects of PKA gain- and loss of function permit a detailed dissection of the organization of cell–cell contacts as driven by Moody/PKA signaling and allow us to track its role in the continued growth of the SPG during larval stages. We observe asymmetric and opposing subcellular distributions of Moody and PKA, providing novel insight into the establishment of apical–basal polarity in the SPG as a secondary epithelium, as well as its morphogenetic function. We present a 3D reconstruction of SJ ultrastructure using serial section transmission electron microscopy (ssTEM) under different PKA activity levels. This new analysis reveals a strict coupling of total cell contact and SJ areas, but also suggests that it is the continuity of individual SJ segments and not total SJ width that is essential for normal BBB insulation. Altogether, our data reveal a previously unrecognized role of GPCR/PKA in maintaining enormous SPG cell growth and its sealing capability by regulating actomyosin contractility and the proper SJ organization in BBB formation and maturation, which touches the fundamental aspects of remodeling cytoskeletal network spatiotemporally – a common process but with different mechanisms in morphogenesis.

## Results

### PKA is required for Moody-regulated BBB formation

**Figure 1.**
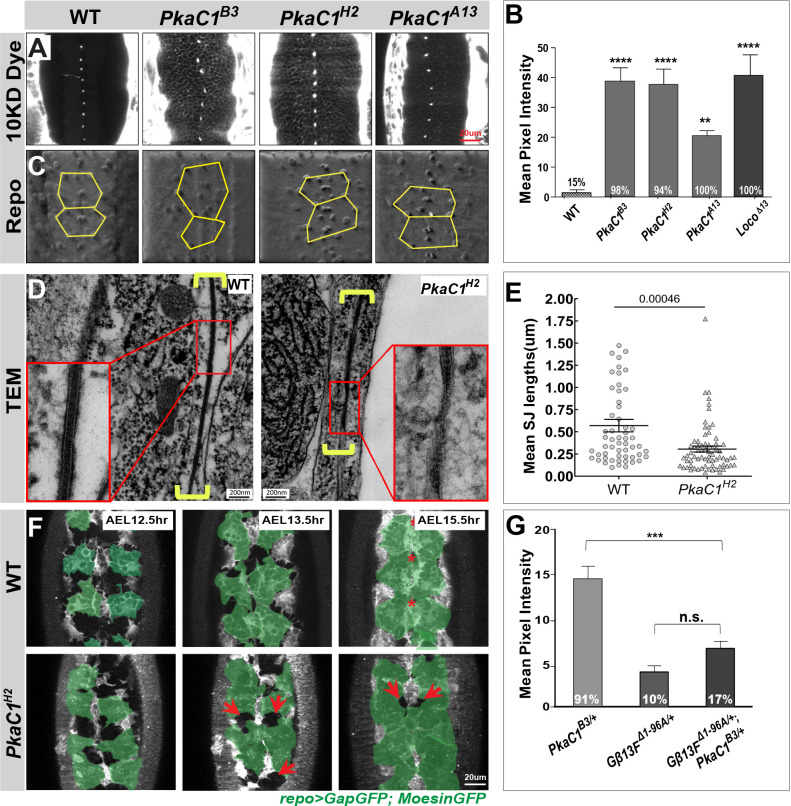
Protein kinase A (PKA) is required for blood–brain barrier (BBB) formation and acts in the Moody signaling pathway. (**A**) Single confocal sections of dye-injected embryos of WT and PKA zygotic mutants. (**B**) Quantification of the dye penetration assay. Columns represent the intensity of dye penetration into the nerve cord as measured by the mean pixel intensity (see Experimental procedures), ± SEM, n = 32, 31, 41, 38, 16 in WT, *PkaC1^B3^, PkaC1^H2^, PkaC1^A13^, Loco^∆13^* embryos, respectively. *Loco^∆13^* zygotic mutants serve as positive controls. (**C**) Repo staining revealing the number and positions of subperineural glia (SPG) nuclei in WT and PKA zygotic mutants using an illuminated projection to highlight the ventral surface of the nerve cord. (**D**) Transmission electron micrographs of the interface of neighboring SPG in late WT and *PkaC1^H2^* zygotic mutant embryos. Yellow brackets delineate the septate junction (SJ) ultrastructure; high magnifications are shown in red boxes. (**E**) Quantification of SJ length in WT and *PkaC1^H2^* mutants (see Experimental procedures). Columns represent mean SJ length as measured in random nerve cord sections, ± SEM, n = 56 and n = 70 in WT and *PkaC1^H2^* mutants, respectively. (**F**) Time-lapse recording of BBB closure in embryos of WT and *PKA* zygotic mutants. 6 µm confocal stacks are shown; in each image, 4–6 ventral SPG are highlighted (green); midline channels (stars) and retarded growth (arrows) are marked. (**G**) Dominant genetic interactions between *PkaC1^B3^* and *Gβ13F^∆1-96A^* as quantified by dye penetration in the embryo. Columns represent the intensity of dye penetration as measured by the mean pixel intensity, ± SEM, n = 34, n = 48, and n = 71 in *PkaC1^B3/+^*, *Gβ13F^∆1-96A/+^*, and *Gβ13F^∆1-96A/+^;PkaC1^B3/+^* mutants, respectively. In (**B**) and (**G**), the percentage of embryos showing the dye penetration is indicated at the bottom of each column. Brackets and asterisks in (**B**), (**E**), and (**G**) indicate statistical significance levels as assessed by ordinary one-way ANOVA with Dunnett’s multiple comparisons test in (**B**) and (**G**) or the two-tailed Student’s t-test in (**E**), n.s., p>0.05; *p<0.05; **p<0.01; ***p<0.001.

To identify molecules that act downstream of Moody signaling in BBB formation, we examined genes known to be involved in GPCR signaling, such as PkaC1, PI3K, PTEN, PLC, and Rap1. We tested BBB permeability in genomic mutants or transgenic RNAi knockdowns of these GPCR effectors by injecting a fluorescent dye into the body cavity and determining its penetration into the CNS using confocal imaging. We found that zygotic mutants of the PKA catalytic subunit PkaC1 (originally named DC0 in *Drosophila*), namely, the two null alleles *PkaC1^B3^* and *PkaC1^H2^* as well as the hypomorphic allele *PkaC1^A13^* (Kalderon and Rubin, 1988), show severe CNS insulation defects (Figure 1A and B), similar in strength to zygotic mutants of the negative regulator *loco*. By contrast, the removal of the other candidates had no effect (data not shown). *PkaC1* has both maternal and zygotic components, and its maternal contribution perdures until late embryogenesis (Lane and Kalderon, 1993). The BBB defect we observe could explain the morphologically inconspicuous embryonic lethality of *PkaC1* zygotic null mutants (Lane and Kalderon, 1993). To rule out the possibility that the observed BBB defects are caused by glial cell fate or migration defects, we examined the presence and position of SPG using an antibody against the pan-glial, nuclear protein Reversed polarity (Repo) (Halter et al., 1995). In *PkaC1* zygotic mutants, the full set of SPG is present on the surface of the nerve cord, although the position of the nuclei is more variable than in WT (Figure 1C), an effect that is also observed in known mutants of the Moody signaling pathway (Granderath et al., 1999; Schwabe et al., 2005).

**Figure 1—figure supplement 1.**
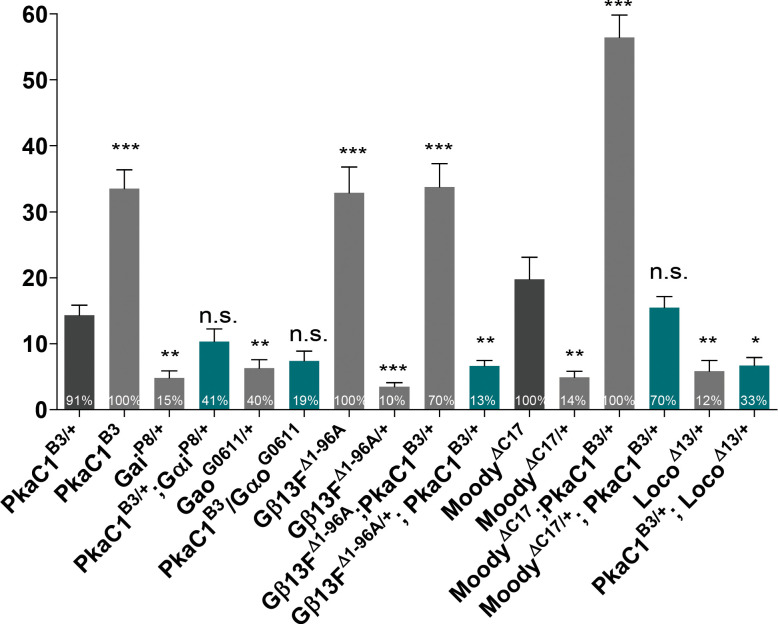
Genetic interactions between protein kinase A and different Moody pathway components. Quantitative analysis of dye penetration shows that the mild blood–brain barrier defect of *PkaC1^B3^* heterozygotes can be partially rescued by removing one copy of Gβ13F as well as one copy of Loco. The removal of one copy of other Moody pathway components, such as moody, Gαo, and Gαi, results in no or weaker, non-significant rescue, suggesting that Gβ13F is more dosage sensitive. Asterisks indicate statistical significance levels as assessed by one-way ANOVA with Dunnett’s multiple comparisons test, n.s., p>0.05; *p<0.05; **p<0.01; ***p<0.001.

**Figure 1—video 1.** Subperineural glia (SPG) epithelium formation in WT embryos. The SPG (labeled by the membrane marker GapGFP and the actin marker MoesinGFP, driven by the pan-glial driver *repoGAL4*) are fairly uniform in size and cell shape, and their spreading and cell–cell contact formation are highly synchronized. The glial sheet is closed by 15.5 hr of development.

**Figure 1—video 2.** Subperineural glia (SPG) epithelium formation in protein kinase A (PKA) zygotic null mutant embryos. The SPG of *PkaC1^B3^* zygotic mutants show variable size and cell shape, and their spreading and contact formation are poorly synchronized, resulting in patchy cell–cell contacts with gaps of different sizes. Moreover, the complete closure of the SPG epithelium is delayed compared to WT.

Since SJs are the principal structure providing BBB insulation and are disrupted in Moody pathway mutants (Schwabe et al., 2005; Schwabe et al., 2017), we sought to characterize the SJ morphology in PkaC1 mutants. We performed ultrastructural analysis of SJs in late embryos (*after egg lay (AEL)* 22–23 hr) by TEM using high-pressure freezing fixation. In WT, the SJs are extended, well-organized structures that retain orientation in the same plane over long distances (Figure 1D). In contrast, in *PkaC1^H2^* zygotic mutants, the overall organization of SJs appears perturbed, and their length, as measured in random single sections, is significantly shorter than in WT (0.31 ± 0.03 µm vs. 0.57 ± 0.07 µm, p=0.000457; Figure 1D and E); very similar phenotypic defects are observed in *moody* and *loco* zygotic mutants (Schwabe et al., 2005).

To explore the role of PkaC1 during development of the BBB, we performed time-lapse recordings of SPG epithelium formation. The SPG arise in the ventrolateral neuroectoderm and migrate to the surface of the developing nerve cord (Ito et al., 1995), where they spread until they reach their neighbors and form intercellular SJs (Schwabe et al., 2005; Schwabe et al., 2017). To monitor the changes in SPG morphology during the closure process, we expressed the membrane marker *GapGFP* and the actin marker *MoesinGFP* using the pan-glial driver *repoGAL4* (Schwabe et al., 2017; Figure 1, Figure 1—video 1, Figure 1—video 2). In WT embryos, SPG are relatively uniform in cell size and shape, and grow to form cell–cell contacts in a highly synchronized manner. By 15.5 hr of development, the glial sheet is closed (Figure 1F). By contrast, SPG in *PkaC1^H2^* zygotic mutants show increased variability in size and shape, and their spreading and contact formation is less well coordinated. This results in patchy cell–cell contacts with gaps of variable sizes (Figure 1F). Moreover, the complete closure of the SPG epithelium is delayed compared to WT (Figure 1F). Again, the defects observed in PKA loss of function are similar to those in Moody pathway mutants (Schwabe et al., 2017).

Our results show that *PkaC1* is required for BBB integrity, proper SJ organization, and SPG epithelium formation, in all cases closely mimicking the phenotypes observed for known Moody signaling components. Given these similarities, we sought to determine whether PKA participates in the Moody pathway by performing dominant genetic interaction experiments. Notably, we found that embryos heterozygous for *PkaC1* null alleles, which are known to have ~50% of wildtype PkaC1 activity, show mild BBB permeability defects (Figure 1G). Therefore, we used *PkaC1^B3^* heterozygous mutants as a sensitized genetic background and removed one genomic copy of different Moody pathway components, including Moody, Loco, Gαo, Gαi, and Gβ13F (Schwabe et al., 2005), to determine whether any synergistic or antagonistic interactions are observed. We found that the dye penetration defects of *PkaC1* heterozygous mutants are significantly reduced by removing one genomic copy of *Gβ13F* or *loco* (Figure 1G and Figure 1—figure supplement 1, p=0.0022 or p=0.0115); removal of one genomic copy of *Gβ13F* or *loco* on their own has no effect. These genetic interactions indicate that PkaC1 is indeed part of the Moody signaling pathway. Removal of single copies of other pathway components showed either a mild, non-significant or no effect in a *PkaC1^B3^* background, suggesting that they are less dosage-sensitive (Figure 1—figure supplement 1).

### PKA is required for BBB continued growth in larvae and BBB maintenance in adults

**Figure 2.**
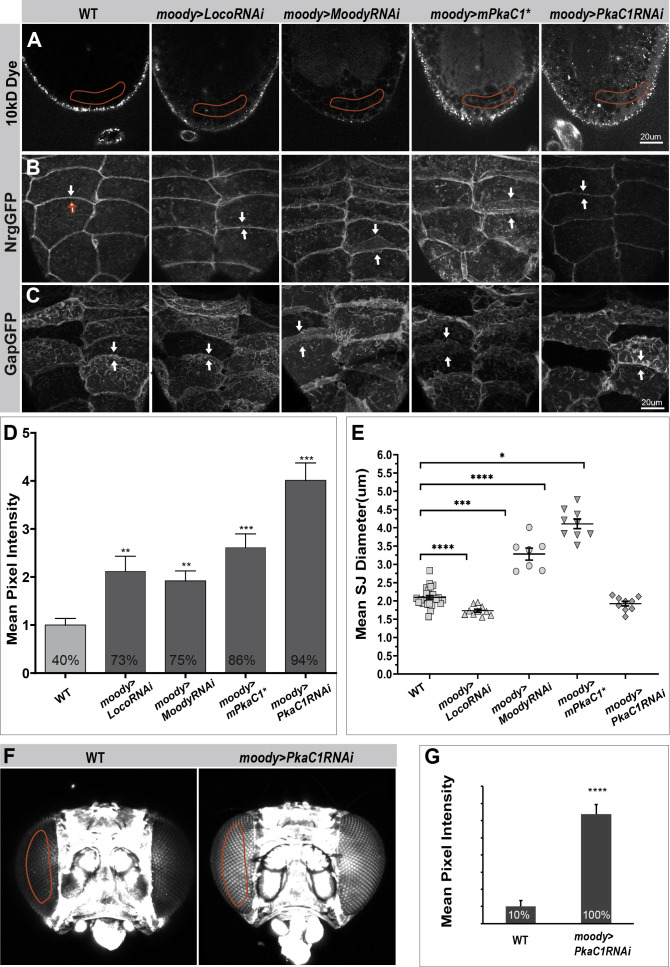
Moody/protein kinase A (PKA) signaling is required for blood–brain barrier (BBB) growth in the larva and for BBB maintenance in the adult. (**A**) Single confocal sections of dye-injected third instar larval nerve cords under different Moody/PKA activity levels. (**B, C**) Morphology of subperineural glia (SPG) septate junction (SJ) belts and membrane overlap at different Moody/PKA activity levels, as visualized by SJ markers NrgGFP (**B**), and the membrane marker GapGFP (**C**). (**D**) Quantification of the dye penetration assay. Columns represent intensity of dye penetration as measured by mean pixel intensity (see Experimental procedures), ± SEM, n = 44–88. The percentage of larva showing dye penetration is indicated at the bottom of each column. (**E**) Quantification of the diameter of SJ belts under different GPCR/PKA activity levels, using the SJ marker NrgGFP.± SEM, n = 7–28. (**F**) Dye penetration in adult flies as shown in z-projections of dye-injected adult heads. (**G**) Quantification of dye penetration in adult eye. Columns represent intensity of dye penetration as measured by mean pixel intensity in each adult eye (see Experimental procedures), ± SEM, n = 30 and 18. Asterisks in (**D**), (**E**), and (**G**) indicate significance levels of comparisons based on Welch’s ANOVA with Dunnett’s T3 multiple comparisons test (**D**) and (**E**) or the two-tailed unpaired t-test (**G**), n.s., p>0.05; *p<0.05; **p<0.01, ***p<0.001, ****p<0.0001.

For a more detailed analysis of PkaC1 function in BBB regulation, we turned to the SPG epithelium in third instar larvae. During the larval stage, no additional SPG cells are generated, instead the existing SPG cells grow enormously in size to maintain integrity of the BBB (Li et al., 2017; Unhavaithaya and Orr-Weaver, 2012). By third instar, they have roughly doubled in size and are accessible via dissection of the CNS, which greatly facilitates the microscopic analysis. PKA activity in larvae can be manipulated specifically using the SPG-specific driver *moodyGAL4* (Bainton et al., 2005; Schwabe et al., 2005), which becomes active only after epithelial closure and BBB sealing are completed in stage 17 embryos. PKA can be reduced by expression of transgenic RNAi targeting the PKA catalytic subunit C1 (*moody>PkaC1* RNAi). On the other hand, PKA can be elevated by expression of a mouse constitutively active PKA catalytic subunit (*moody*>mPkaC1*; Zhou et al., 2006). We first examined whether normal Moody/PKA activity is required for BBB integrity during larval stages. To address this question, we developed a dye penetration assay to measure BBB permeability in cephalic complexes of third instar larval. This assay is similar to the one we performed in the late embryo, but with some important modifications (for details, see Experimental procedures). Interestingly, both elevated and reduced activity of Moody (*moody>LocoRNAi* and *moody>moodyRNAi*) and PKA (moody>mPKAC1* and *moody>PKAC1* RNAi) in SPG resulted in severe BBB insulation defects (Figure 2A and D). This strongly suggests that Moody/PKA signaling plays a crucial role in the continued growth of the BBB during larval stages. These effects were not merely carried over from the embryo since under *moody* driver caused only mild dye penetration defects in embryos (Figure 2—figure supplement 1). Given that Moody activity has been implicated in the maintenance of the BBB in the adult (Bainton et al., 2005), we also sought to knockdown PKA specifically in the adult SPG (*tubGal80ts, moody>PkaC1* RNAi) and measure the resulting effects (see Experimental procedures). We observed the dye significantly penetrated the blood–eye barrier under reduced PKA expression compared to that in WT (1.00 ± 0.34 vs. 6.38 ± 0.37, p<0.000001; Figure 2F and G), indicating that PKA is indeed also required for BBB integrity function in the adult.

**Figure 2—figure supplement 1.**
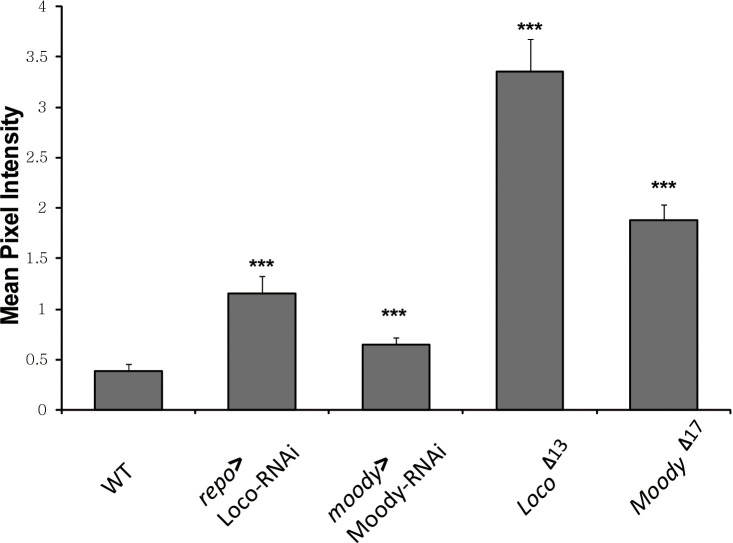
Dye penetration defects for RNAi vs. genomic mutants in the embryo. Graph shows quantification of penetration of fluorescent dye into the CNS in WT, loco, and moody null alleles, as well as loco or moody-RNAi expressed under repo- or moody-Gal4; 22 hr embryos. While moody-RNAi expressed using moody-Gal4 results in measurable dye penetration, the defect is much milder than in the mutant. Asterisks indicate statistical significance levels as assessed by one-way ANOVA with Dunnett’s multiple comparisons test, n.s., p>0.05; *p<0.05; **p<0.01; ***p<0.001.

**Figure 2—figure supplement 2.**
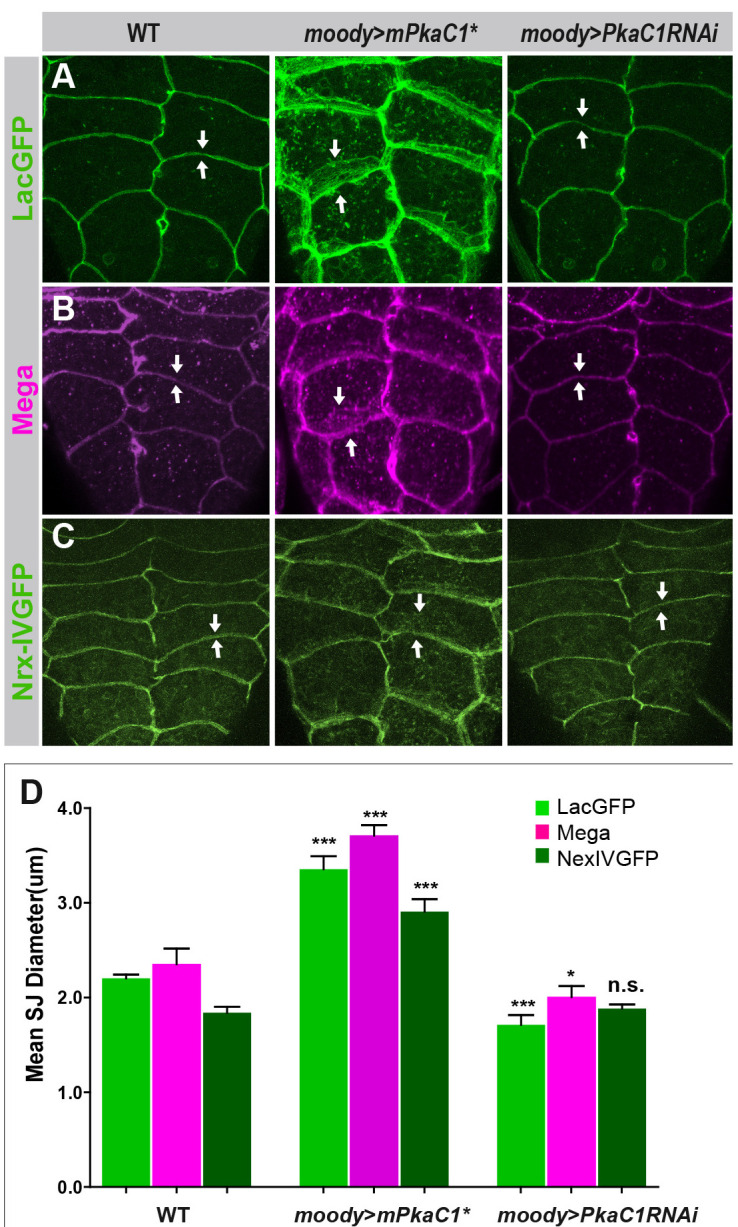
Morphology of subperineural glia (SPG) septate junction (SJ) belts at different protein kinase A (PKA) activity levels, as visualized by SJ markers LacGFP (**A**), Mega (**B**), and Nrx-IVGFP (**C**). (**D**) Quantification of the diameter of SJ belts under different PKA activity levels, using different SJ markers. All groups in PKA overactivity and one group of LacGFP labeled SJ belt in PKA underactivity are significantly different from WT, as assessed by Welch’s ANOVA with Dunnett’s T3 multiple comparisons test, n.s., p>0.05; *p<0.05; **p<0.01, ***p<0.001.

In order to better understand the cause of BBB permeability under conditions where Moody/PKA is changed, we examined SJ morphology in larvae. Most core SJ components show interdependence for correct localization and barrier function, with removal of one component sufficient to abolish SJ function (Behr et al., 2003; Genova and Fehon, 2003; Hijazi et al., 2011; Oshima and Fehon, 2011; Wu et al., 2004). We therefore asked whether PKA activity levels affect the distribution of different SJ components. Using both live imaging (*NrgGFP, LacGFP, NrxIVGFP*) and immunohistochemistry (Mega), we found that the circumferential SJ belts and outlines of SPG were marked nicely in WT (Figure 2B and Figure 2—figure supplement 2). Strikingly, upon either reduction of Moody activity or elevated PKA activity, the SJ belt staining became much broader and more diffuse than in WT (Figure 2B). This suggests extensive plasma membrane overlap between neighboring SPG cells. To confirm this idea, we introduced the membrane marker *gapGFP*, and indeed observed increased membrane overlap compared to WT (Figure 2C). Conversely, both elevated Moody activity and reduced PKA activity resulted in thinner SJ belts and reduced membrane contacting area (Figure 2B and C). To quantify these changes, we measured the mean width of the SJ belts under different PKA activity levels (Figure 2E; Experimental procedures). The mean width of SJ belts increased with elevated PKA activity/reduced Moody activity and decreased under inverse conditions compared to WT (Figure 2E). These data demonstrate that Moody and PKA are required for the continued growth of the BBB and the proper organization of SJs during larval stages. Unlike the barrier defect, these morphological data reveal a monotonic relationship between PKA activity, membrane overlap, and the amount of SJ components in the area of cell contact. The fact that the cellular defects of reduced Moody activity match those of elevated PKA activity, and vice versa, provides further evidence that PKA acts as an antagonistic effector of Moody signaling.

### PKA regulates the cytoskeleton and vesicle traffic in SPG

We had previously reported that the Moody pathway regulates the organization of cortical actin and thus the cell shape of SPG during late embryogenesis (Schwabe et al., 2005; Schwabe et al., 2017). Moreover, we proposed, based on the developmental timeline, that this in turn affects the positioning of SJ material along the lateral membrane. Given that the most striking phenotype caused by altered PKA activity is the extent of membrane overlap, we sought to further explore if PKA functions by regulating the cytoskeleton in SPG.

**Figure 3.**
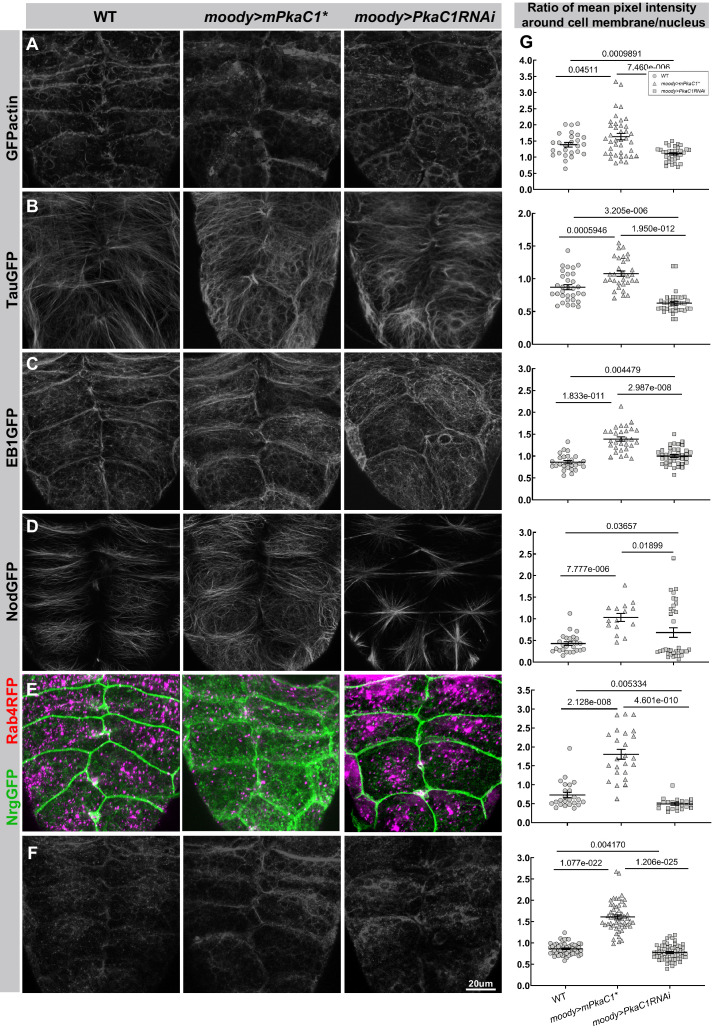
Protein kinase A (PKA) regulates the cytoskeleton and vesicle distribution in subperineural glia (SPG). Under different PKA activity levels, the actin cytoskeleton is visualized by GFPactin (**A**), the microtubule cytoskeleton by the general MT marker TauGFP (**B**), the plus-end marker EB1GFP (**C**), and the minus-end marker NodGFP (**D**), the cellular distribution of vesicles by the early endosome markers Rab4RFP (**E**) and Rab11GFP (**F**) with the septate junction (SJ) marker NrgGFP labeling the cell periphery of the SPG (**E**). (**G**) Quantification of the distribution of all markers around membrane area and the nucleus area under different PKA activity levels, including GFPactin, TauGFP, EB1GFP, NodGFP, Rab4RFP, and Rab11GFP. Columns represent the ratio of mean pixel intensity around cell membrane/nucleus in random SPG cells, ± SEM, n = 22–54. Asterisks indicate significance levels of comparisons based on Brown–Forsythe and Welch’s ANOVA with multiple comparisons test, p value is label in each comparison group. A strong cortical actin rim around cell periphery is visible in WT (**A**). Compared with WT, the cortical actin rim is wider and stronger upon increased PKA activity and becomes weaker and less regular with reduced PKA activity. In WT (**B**–**D**), TauGFP-labeled microtubule fibers extend from MTOC to cell periphery throughout the cytoplasm (**B**); EB1GFP is enriched at the cell cortex but visible throughout the cytoplasm (**C**); NodGFP is enriched on fibers around the nucleus, but not around the cell cortex (**D**). Upon increased PKA activity, EB1GFP shows broader and more diffuse localization at the cell cortex, TauGFP and NodGFP become enriched at the cell periphery and show a web-like structure throughout the cytoplasm. Upon reduced PKA activity, TauGFP reveals disorganized and wavy microtubule organization; EB1 localization at the cell cortex is reduced; NodGFP accumulates around the MTOC in a striking star-shaped fashion. Rab4RFP- and Rab11GFP-labeled endosomes are differentially enriched in the cell periphery under PKA overactivity and surrounding the nucleus under reduced PKA activity when compared to its broader pan-cytoplasmic distribution in WT (**E, F**).

For this purpose, we examined the intracellular distribution of the actin cytoskeleton in the SPG at different PKA levels. As live markers, we used *GFPactin*, which labels the entire actin cytoskeleton, *RFPmoesin* (Schwabe et al., 2005), that preferentially labels the cortical actin, the presumptive general MT marker *TauGFP* (Jarecki et al., 1999), the plus-end marker *EB1GFP* (Rogers et al., 2004), and the minus-end marker *NodGFP* (Clark et al., 1997; Cui et al., 2005; Figure 3A–D, and data not shown). In response to changes in PKA activity, all markers showed altered distributions similar to those observed with SJ markers. Specifically, elevated PKA activity caused all markers to become enriched at the cell cortex area, consistent with the broader membrane contacting area between neighboring SPG (Figure 3A–D, middle column). Conversely, upon reducing PKA activity, all markers were reduced or depleted from the cell cortex, consistent with reduced contact area between neighboring SPG (Figure 3A–D, right column). Thus, PKA signaling profoundly reorganizes the actin and MT cytoskeleton, which may affect the membrane contacting area between neighboring SPG.

Since PKA has been shown to affect vesicle trafficking in epithelial cells and neurons (Renger et al., 2000; Vasin et al., 2014; Wojtal et al., 2008; Zhang et al., 2007), we investigated if PKA signaling has a similar role during continued SPG cell growth. We introduced two live markers, *Rab4RFP*, which labels all the early endosomes (Figure 3E), and *Rab11GFP* (Artiushin et al., 2018), which labels both early and recycling endosomes (Figure 3F). We observed significant changes in the cellular distribution of vesicle populations. Specifically, Rab4- and Rab11-labeled endosomes were differentially enriched in the cell periphery when PKA activity is increased and surrounded the nucleus when PKA was reduced, as compared to their broader cytoplasmic distribution profile in WT (Figure 3E and F). Therefore, our results with these different cytoskeletal and vesicle markers suggest that the major function of PKA is regulating the cell contact areas between neighboring SPGs: high levels give rise to broad membrane contacts, and low levels of PKA activity cause narrow membrane contacts, which then affects SJ organization and BBB function.

### The continuity of SJ belt is essential for BBB function as revealed by ssTEM

**Figure 4.**
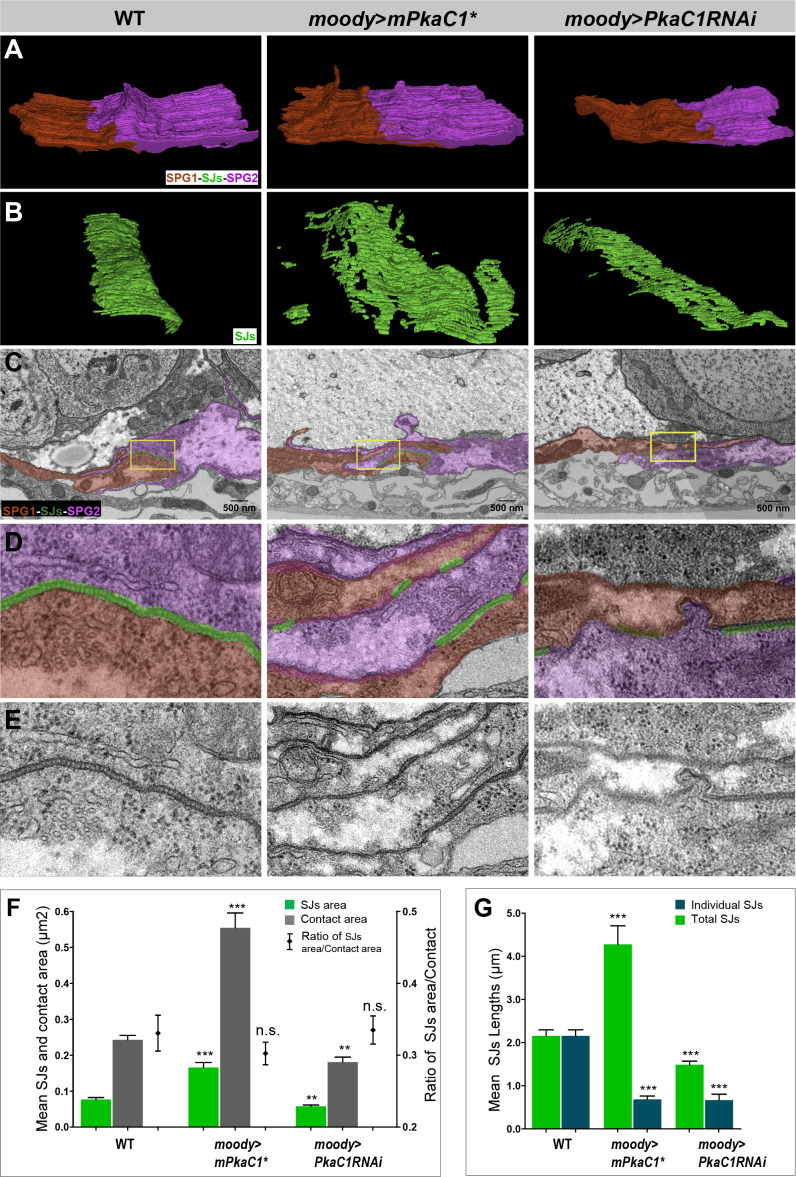
The continuity of the septate junction (SJ) belt is essential for blood–brain barrier (BBB) function as revealed by serial section transmission electron microscopy (ssTEM). (**A–E**) SJ ultrastructure at the interface of neighboring subperineural glia (SPG) in third instar larvae under different protein kinase A (PKA) activity levels. SPG1, its neighbor SPG2, and their shared SJs are colored or shaded in red, magenta, and green, respectively. (**A, B**) A 3D model of SJ ultrastructure generated by ssTEM. (**C**) Representative sections of SJs. (**D, E**) High-magnification views of boxed regions in (**C**) with and without shading. In WT, the area of contacting SPG is compact and well defined, and a dense SJ belt is formed between neighboring SPG. Under PKA overactivity (moody>mPkaC1*), neighboring SPG show much deeper interdigitations, and the SJs belt are discontinuous and appear patchy. Under PKA underactivity (*moody>PkaC1* RNAi), the cell contact and SJ area are reduced, and the SJ belt becomes patchy too. (**F**) Quantification of SJ surface area (green column) and the contact area (grey column), and the ratio between these two area (black point) under different PKA activity levels, ± SEM, n = 15–21. (**G**) Quantification of the mean length of individual SJ segments (green) and the mean total length of SJs (blue) under different PKA activity levels, measured in random nerve cord sections, ± SEM, n = 9–92. Asterisks in (**F, G**) indicate significance levels of comparisons based on Welch’s ANOVA with Dunnett’s T3 multiple comparisons test, n.s., p>0.05; *p<0.05; **p<0.01, ***p<0.001. Compared to WT, the mean total length of SJs significantly increases (4.28 ± 0.43 µm vs. 2.16 ± 0.14 µm, p=0.000523, about twofold) and the mean length of individual SJ segments significantly decreases (0.69 ± 0.08 µm vs. 2.16 ± 0.14 µm, p<0.0001, about 0.3-fold) under PKA overactivity; both the mean total length of SJs (1.49 ± 0.08 µm vs. 2.16 ± 0.14 µm, p=0.000878, about 0.69-fold) and the mean length of individual SJ segments decrease (0.67 ± 0.14 µm vs. 2.16 ± 0.14 µm, p<0.0001, about 0.31-fold), respectively, upon reduced PKA activity.

While PKA gain- and loss of function show opposite morphologies of membrane overlap and SJ belt by light microscopy, they both result in a compromised leaky BBB. To better understand this incongruence, we sought to analyze membrane morphology at a higher resolution. Due to the small size of SJs (20–30 nm), structural aspects can be analyzed conclusively only by electron microscopy. In the past, the acquisition and analysis of a complete series of TEM sections required an enormous effort; as a consequence, studies of SJ structure have mostly been restricted to random sections (Carlson et al., 2000; Hartenstein, 2011; Stork et al., 2008; Tepass and Hartenstein, 1994). The problem has now become solvable, using digital image recording (Suloway et al., 2005) and specialized software (Fiji, TrakEM2)(Cardona et al., 2012; Schindelin et al., 2012) for both image acquisition and post-processing. Therefore, we performed serial section TEM, followed by computer-aided reconstruction of TEM stacks, to resolve the 3D ultrastructure of cell contacts and SJs under different PKA activity levels at third instar larva (Figure 4A and B). This is the first time that a contiguous SJ belt between neighboring SPG at nanometer resolution is presented.

**Figure 4—figure supplement 1.**
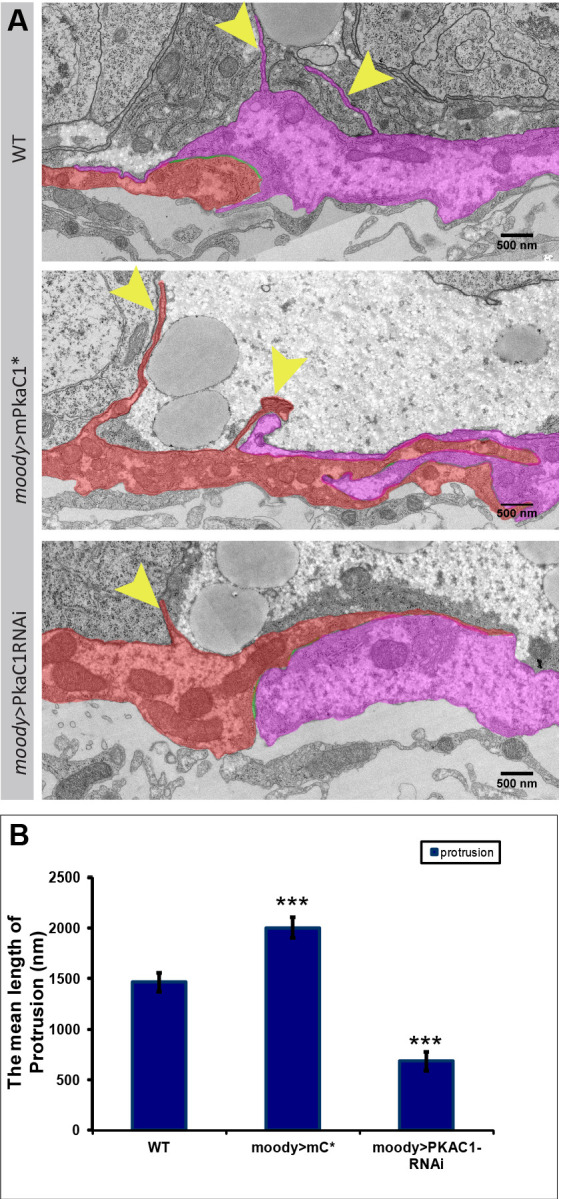
Morphology of apical membrane protrusions of subperineural glia (SPG) at diﬀerent protein kinase A (PKA) activity levels, as revealed by serial section transmission electron microscopy (ssTEM). (**A**) Representative sections of the apical membrane protrusion of SPG ultrastructure under diﬀerent PKA levels, marked by yellow arrows. SPG1, its neighbor SPG2, and their shared SJs are colored in red, magenta, and green, respectively. (**B**) Quantiﬁcation of the mean length of individual apical protrusion under diﬀerent PKA activity levels, measured in random nerve cord sections, ± SEM, n = 14–35. Compared to WT, the mean length of apical membrane protrusion of SPG is signiﬁcantly longer under PKA overactivity and signiﬁcantly shorter under PKA underactivity. Statistical signiﬁcance of comparisons was assessed using Welch’s ANOVA with Dunnett’s T3 multiple comparisons test, n.s., p>0.05; *p<0.05; **p<0.01, ***p<0.001.

In WT, the area of cell–cell contact is compact and well-defined, with a dense SJ belt covering ~30% of the cell contact area (Figure 4A, B and F). Upon elevated PKA activity, neighboring SPG show much deeper membrane overlap (Figure 4A–E). The areas of both cell contact and SJ coverage increase about twofold compared with WT (Figure 4F), confirming the observations from confocal microscopy (Figure 3A–D), but the SJ belt is discontinuous and appears patchy (Figure 4B–E). This suggests that it is the continuity of the belt, rather than the total area covered by SJs, that is essential for generating the intercellular sealing capacity. To examine this question directly, we measured SJ length in randomly selected sections. Compared with WT, the mean length of individual SJ segments (0.69 ± 0.08 µm vs. 2.16 ± 0.14 µm, p<0.0001) is indeed significantly decreased, while the mean total length of SJs (4.28 ± 0.43 µm vs. 2.16 ± 0.14 µm, p=0.000523) is significantly increased (Figure 4G).

Upon reducing PKA activity, the cell contacts and SJ area between neighboring SPGs were reduced, and the SJ belt became patchy as well (Figure 4A–E). In this case, both the mean total length of SJs (1.49 ± 0.08 µm vs. 2.16 ± 0.14 µm, p=0.000878) and the mean length of individual SJ segments (0.67 ± 0.14 µm vs. 2.16 ± 0.14 µm, p<0.0001) were significantly shorter than in WT (Figure 4G). Intriguingly, the ratio of total SJ area to cell contact area remains constant at about 30% under all PKA activity conditions, despite the variable interdigitations between contacting SPG (Figure 4F).

Finally, SPG send apical protrusions into the neural cortex (Figure 4—figure supplement 1). These protrusions are much longer (2.01 ± 0.01 µm vs. 1.47 ± 0.09 µm, p=0.000230) upon elevated PKA activity and shorter than in WT (0.68 ± 0.09 µm vs. 1.47 ± 0.09 µm, p<0.0001) upon reduced PKA activity, suggesting that PKA activity more generally controls membrane protrusions and extension (Figure 4—figure supplement 1).

Taken together, our ultrastructural analyses and new 3D models support the light microscopic findings, and they provide superior quantification of the relevant parameters. Importantly, cell contact and SJ area, as well as total SJ content, are monotonically correlated with PKA activity, while individual SJ segment length is not. This suggests that the discontinuity of the SJ belt is the main cause for the observed BBB permeability defects.

### The Moody/PKA signaling pathway is polarized in SPG

The SPG are very thin cells, measuring around 0.2 µm along the apical–basal axis. In the embryo, the hemolymph-facing basal surface of the SPG is covered by a basal lamina (Fessler et al., 1994; Olofsson and Page, 2005; Tepass and Hartenstein, 1994), while during larval stages, the perineurial glia (PNG) form a second sheath directly on top of the SPG epithelium, which then serves as the basal contact for the SPG (Stork et al., 2008; Stork et al., 2008). Consistent with its chemoprotective function, the Mdr65 transporter localizes to the hemolymph-facing basal surface of the SPG, while Moody localizes to the CNS-facing apical surface (Mayer et al., 2009). The shallow lateral compartment contains the SJs, which not only seal the paracellular space but also act as a fence and prevent diffusion of transmembrane proteins across the lateral compartment. The apical localization of Moody protein is dependent on the presence of SJs (Schwabe et al., 2017).

**Figure 5.**
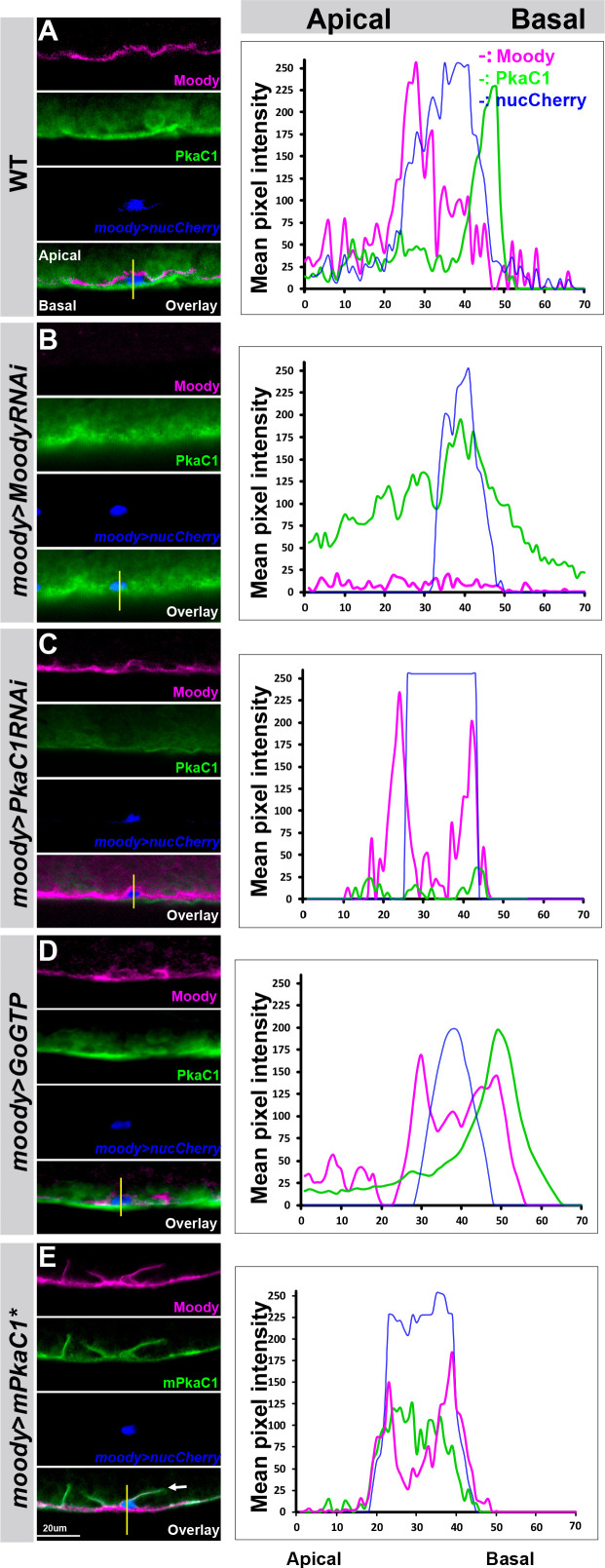
The Moody/protein kinase A (PKA) signaling pathway is polarized in subperineural glia (SPG). The subcellular localization of the PKA catalytic subunit PkaC1 and Moody in SPG of third instar larvae in WT (**A**), Moody knockdown (*moody>MoodyRNAi*) (**B**), PkaC1 knockdown (*moody*>Pka-C1-RNAi), GPCR gain of function (*moody*>Go GTP), and PKA overexpression (*moody*>mPka-C1*). Antibody labeling of Moody (magenta), of *Drosophila* PkaC1 or mouse PkaC1 (green), and of SPG nuclei (*moody>nucCherry*; blue). (**A–E**) Lateral views of the CNS/hemolymph border, with CNS facing top. On the right column, line scans of fluorescence intensities for each channel along the apical–basal axis at the positions indicated. In WT (**A**), Moody localizes to the apical side and PkaC1 is enriched at the basal side of SPG. Under loss of Moody signaling (*moody>MoodyRNAi*) (**B**), PKA spreads throughout the cell and loses its basal localization. Moody loses its apical localization under reduced (**C**) or increased PKA activity (**E**). Under GPCR gain of function (**D**), Pka-C1 is basally polarized, while Moody lost its asymmetric localization in SPG.

To visualize the subcellular protein distributions along the apical–basal axis, we labeled them together with the SPG nuclei (*moody>nucCherry*). We examined the subcellular distribution of PkaC1 by immunohistochemistry (anti-PKA catalytic subunit antibody, which only bind to the catalytic subunits of PKA dissociated from the regulatory subunits of PKA after cAMP activation, not binding to the inactive enzyme) and found that active PkaC1 is enriched on the basal side of the SPG, and thus the opposite of the apically localized Moody (Figure 5A). This result is intriguing given PKA’s antagonistic role in Moody signaling and suggests that pathway activity may affect the localization of pathway components.

We further examined the subcellular distribution of Moody and Pka-C1 in gain- or loss-of-function conditions of Moody/PKA signaling. Notably, the subcellular distribution of PkaC1 was indeed altered when Moody is knocked down in SPG (moody>Moody RNAi). PkaC1 lost its basal intracellular localization and appeared spread out throughout the cytoplasm (Figure 5B), suggesting that Moody is required for Pka-C1 polarized localization. Upon reduced PKA activity, Moody loses its apical localization (Figure 5C). Meanwhile, upon increased PKA activity, Pka-C1 appears at both sides of SPG as expected (Figure 5E), and Moody loses its apical membrane localization and is found at both the apical and basal membranes of SPG (Figure 5E), suggesting that PKA activity affects the subcellular localization of Moody. In addition, under gain of function of Moody signaling (moody>Gαo-GTP), PKA remains basally localized (Figure 5D), regardless of Moody’s mis-localization, suggesting that Gαo signaling is sufficient for PKA enrichment at the basal side of SPG. Given the profound effect of PKA activity on the organization of SJ belt (Figure 2B and D), the cytoskeleton architecture and polarity, as well as vesicle transport (Figure 3A–F), mis-localized Moody could be an effect of deregulated protein trafficking or dysfunction of SJs, which normally restrict the diffusion of molecules between membrane compartments. Taken together, our data suggest that apical Moody signaling is necessary for repressing apical PkaC1 protein accumulation, and that this polarized subcellular localization results from the antagonistic relationship between Moody and PKA.

### MLCK and Rho1 function as PKA targets in the SPG

**Figure 6.**
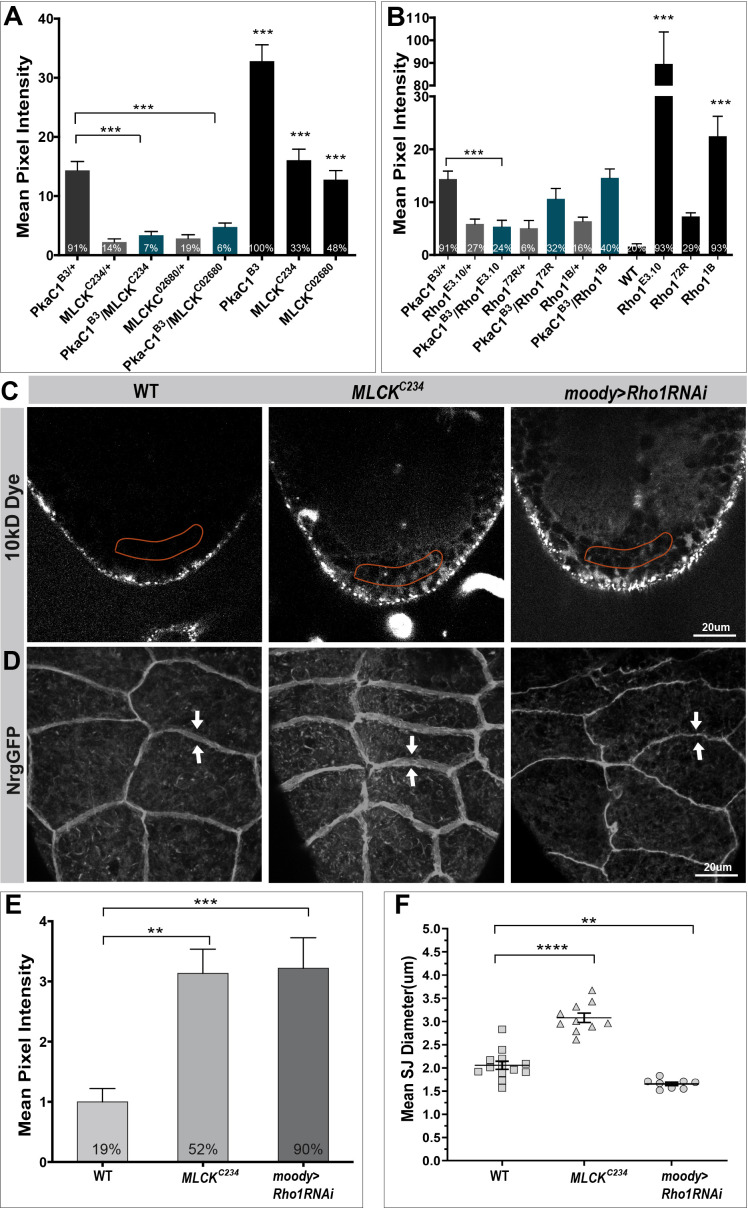
Myosin light chain kinase (MLCK) and Rho1 function as protein kinase A (PKA) targets in subperineural glia (SPG). (**A**) Quantification of dye penetration effects in the embryo of *MLCK* and *Rho1*. (**B**) Dominant genetic interactions between *PkaC1^B3^* and *MLCK* and *Rho1* mutant heterozygotes as quantified by dye penetration in the embryo. In (**A, B**), columns represent the strength of dye penetration into the nerve cord as measured by the mean pixel intensity, ± SEM, n = 14–98. (**C, D**) Blood–brain barrier (BBB) phenotype of *MLCK* zygotic mutant and SPG-specific Rho1 knockdown (*moody>Rho1* RNAi) animals in single confocal sections of dye injected third instar larvae (**C**), and septate junction (SJ) morphology using the NrgGFP marker (**D**), with width of SJ belt highlighted by arrows. (**E**) Quantification of the dye penetration assay from (**C**). Columns represent intensity of dye penetration as measured by mean pixel intensity and normalized to WT mean (see *Materials and methods*, ± SEM, n = 13–19). (**F**) Quantification of the mean diameter of SJ belts from (**D**), ± SEM, n = 8–13. In (**A**), (**B**), and (**E**), the percentage of animals showing dye penetration is indicated at the bottom of each column. Asterisks in (**A**), (**B**), (**E**), and (**F**) indicate significance levels of comparisons against either WT in (**E**) and (**F**) or *PkaC1^B^*^3^ group in (**A**) and (**B**) based on Brown–Forsythe and Welch’s ANOVA with multiple comparisons test, n.s., p>0.05; *p<0.05; **p<0.01, ***p<0.001.

Considering that the most pronounced effect of increasing PKA levels in the SPG is a commensurate increase in membrane overlap at the basolateral side, we sought to genetically identify PKA targets involved in this process. PKA is known to regulate actomyosin contractility by phosphorylating and inhibiting myosin light chain kinase (MLCK), which leads to a decrease in Myosin light chain (MLC) phosphorylation and a concomitant reduction of actomyosin contractility in cell migration and endothelial barrier (Garcia et al., 1995; Garcia et al., 1997; Howe, 2004; Tang et al., 2019; Verin et al., 1998). To determine whether MLCK is required for BBB function, we examined two MLCK zygotic mutants, *MLCK^02860^* and *MLCKC^234^*, and detected moderate BBB permeability in the late embryo (Figure 6A and Figure 6—figure supplement 1), indicating that MLCK plays a role in CNS insulation. Next, we asked whether PKA and MLCK function in the same signaling pathway using dominant genetic interaction experiments. We found that the BBB permeability of *PkaC1^B3^* heterozygous mutants could be rescued by removing one parental copy of *MLCK* (*MLCK^02860^* or *MLCK^C234^*; Figure 6B). This suggests that MLCK interacts with PkaC1 in the SPG. Finally, we examined BBB insulation and SJ defects of *MLCK* zygotic mutant larva (*MLCK^C234^*). *MLCK^C234^* mutant larvae showed significant BBB permeability and a widened SJ belt (Figure 6C–F) compared to WT (Figure 2B), but the phenotypes were milder than those of PKA overactivity (Figure 2B).

**Figure 6—figure supplement 1.**
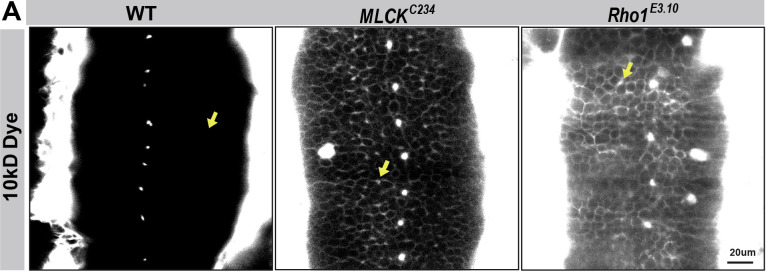
Myosin light chain kinase (MLCK) and Rho1 are required for blood–brain barrier (BBB) integrity in embryos. Single confocal section of dye-injected MLCKC234 zygotic mutant (*MLCK^C234^*), and Rho1 zygotic null mutant (*Rho1^E3.10^*) embryos, with dye penetrating into the nerve cord (yellow arrow) (**A**).

PKA is also known to phosphorylate and inhibit the small GTPase Rho1, which reduces the activity of its effector Rho kinase (ROK), ultimately resulting in decreased MLC phosphorylation and actomyosin contractility (Dong et al., 1998; Garcia et al., 1999; Lang et al., 1996; Tang et al., 2019; Xu and Myat, 2012). Moreover, RhoA activity has been shown to drive actin polymerization at the protrusion of migrating cells (Machacek et al., 2009), and a PKA-RhoA signaling has been suggested to act as a protrusion-retraction pacemaker at the leading edge of the migrating cells (Tkachenko et al., 2011). To check if Rho1 is required for BBB function, we determined the BBB permeability in the late embryo and third instar larval stages. Two loss-of-function alleles, the hypomorphic allele *Rho1^1B^* (Magie and Parkhurst, 2005) and the null allele *Rho1^E.3.10^*, showed dye penetration defects as homozygous zygotic mutant embryos, with the null allele showing a particularly pronounced effect (Figure 6B and Figure 6—figure supplement 1). At the larval stage, the SPG-specific Rho1 knockdown (*moody>Rho1* RNAi) resulted in strong dye penetration into the nerve cord (Figure 6C and E). These results suggest that Rho1 is required for the formation and continued growth of the BBB. We again asked whether PKA and Rho1 function in the same pathway and performed dominant genetic interaction experiments using a sensitized genetic background. The embryonic dye penetration defects of PkaC1 heterozygous mutants (*PkaC1^B3^*) were significantly reduced by removing one genomic copy of the Rho1 null allele (*Rho1^E.3.10^*), but not by removing one copy of the hypomorphic allele *Rho1^1B^* (Figure 6B). These findings suggest that Rho1 is a PKA target in BBB regulation. Collectively, our results indicate that PKA suppresses actomyosin contractility in a two-pronged fashion by negatively regulating both MLCK and Rho1.

## Discussion

Previous studies implicated a novel GPCR signaling pathway in the formation of the *Drosophila* BBB in late embryos (Bainton et al., 2005; Schwabe et al., 2005). This work also revealed that besides the GPCR Moody two heterotrimeric G proteins (Gαiβγ, Gαoβγ) and the RGS Loco participate in this pathway. Here we provide a comprehensive molecular and cellular analysis of the events downstream of G protein signaling using a candidate gene screening approach. We present new, more sensitive methods for phenotypic characterization and extended the analysis beyond the embryo into larval stages. This work identifies PKA, together with some of its targets, as crucial antagonistic effectors in the continued cell growth of SPG and maintenance of the BBB sealing capacity. This role is critical to ensure proper neuronal function during BBB formation and maturation.

Multiple lines of evidence demonstrate a role of PKA for proper sealing of the BBB: loss of PKA activity leads to BBB permeability defects, irregular growth of SPG during epithelium formation, reduced membrane overlap, and a narrower SJ belt at SPG cell–cell contacts. The role of PKA as an effector of the Moody signaling pathway is further supported by dominant genetic interaction experiments, which show that the dye penetration phenotype of *PkaC1* heterozygous mutant embryos was partially rescued by removing one genomic copy of *G*β*13F* or *loco*. Moreover, the analysis of the larval phenotype with live SJ and cytoskeleton markers shows that PKA gain of function behaved similarly to Moody loss of function. Conversely, PKA loss of function resembled the overexpression of GαoGTP, which mimics Moody gain-of-function signaling.

Our results from modulating PKA activity suggest that the total cell contact and SJ areas are a major function of PKA activity: low levels of activity cause narrow contacts, and high levels give rise to broad contacts. Moreover, the analysis of various cellular markers (actin, microtubules, SJs, vesicles) indicates that the circumferential cytoskeleton and delivery of SJ components respond proportionately to PKA activity. This, in turn, promotes the changes in cell contact and junction areas coordinately at the lateral side of SPG. Our experiments demonstrate that the modulation of the SPG membrane overlap by PKA proceeds, at least in part, through the regulation of actomyosin contractility, and that this involves the phosphorylation targets MLCK and Rho1. This suggests that crucial characteristics of PKA signaling are conserved across eukaryotic organisms (Bauman et al., 2004; Marks and Kalderon, 2011; Park et al., 2000; Taylor et al., 1990).

At the ultrastructural level, our ssTEM analysis of the larval SPG epithelium clarifies the relationship between the inter-cell membrane overlaps and SJ organization and function. Across different PKA activity levels, the ratio of SJ areas to the total cell contact area remained constant at about 30%. This proportionality suggests a mechanism that couples cell contact with SJ formation. The primary role of Moody/PKA appears in this process to be the control of membrane contacting area between neighboring cells. This is consistent with the results of a temporal analysis of epithelium formation and SJ insertion in late embryos of WT and Moody pathway mutants, which shows that membrane contact precedes and is necessary for the appearance of SJs (Schwabe et al., 2017). The finding that the surface area that SJs occupy did not exceed a specific ratio, irrespective of the absolute area of cell contact, suggests an intrinsic, possibly steric limitation in how much junction can be fitted into a given cell contact space. While most phenotypic effects are indeed a major function of Moody and PKA activity, the discontinuity and shortening of individual SJ strands is not. It occurred with both increased and decreased signaling and appears to cause the leakiness of the BBB in both conditions. Our ssTEM-based 3D reconstruction thus demonstrates that the total area covered by SJs and the length of individual contiguous SJ segments are independent parameters. The latter appears to be critical for the paracellular seal, consistent with the idea that Moody plays a role in the formation of continuous SJ stands.

The asymmetric localization of PKA that we observed sheds further light on the establishment and function of apical–basal polarity in the SPG epithelium. Prior to epithelium formation, contact with the basal lamina leads to the first sign of polarity (Schwabe et al., 2017). Moody becomes localized to the apical surface only after epithelial closure and SJ formation, suggesting that SJs are required as a diffusion barrier and that apical accumulation of Moody protein is the result of polarized exocytosis or endocytosis (Schwabe et al., 2017). Here, we now show that the intracellular protein PKA catalytic subunit-PkaC1 accumulates on the basal side of SPG, and that this polarized accumulation requires (apical) Moody activity. Such an asymmetric, activity-dependent localization has not previously been described for PKA in endothelium, and while the underlying molecular mechanism is unknown, the finding underscores that generating polarized activity along the apical–basal axis of the SPG is a key element of Moody pathway function.

An intriguing unresolved question is how increased SPG cell size and SJ length can keep up with the expanding brain without disrupting the BBB integrity during larva growth. We found that the SJ grows dramatically in length (0.57 ± 0.07 µm vs. 2.16 ± 0.14 µm, about 3.7-fold) from the late embryo (Figure 1E) to third instar larva (Figure 4G), which matches the increased cell size of SPG (about fourfold; Babatz et al., 2018; Unhavaithaya and Orr-Weaver, 2012). During the establishment of the SPG epithelium in the embryo, both increased and decreased Moody signaling resulted in asynchronous growth and cell contact formation along the circumference of SPG, which in turn led to irregular thickness of the SJ belt (Schwabe et al., 2017). Therefore, a similar relationship may exist during the continued growth of the SPG epithelium in larvae, with the loss of continuity of SJ segments in Moody/PKA mutants resulting from unsynchronized expansion of the cell contact area and an ensuing erratic insertion of SJ components. Since SJs form relatively static complexes, any irregularities in their delivery and insertion may linger for extended periods of time (Babatz et al., 2018; Deligiannaki et al., 2015; Oshima and Fehon, 2011). The idea that shortened SJ segments are a secondary consequence of unsynchronized cell growth is strongly supported by our finding that disruption of actomyosin contractility in MLCK and Rho1 mutants compromises BBB permeability.

**Figure 7.**
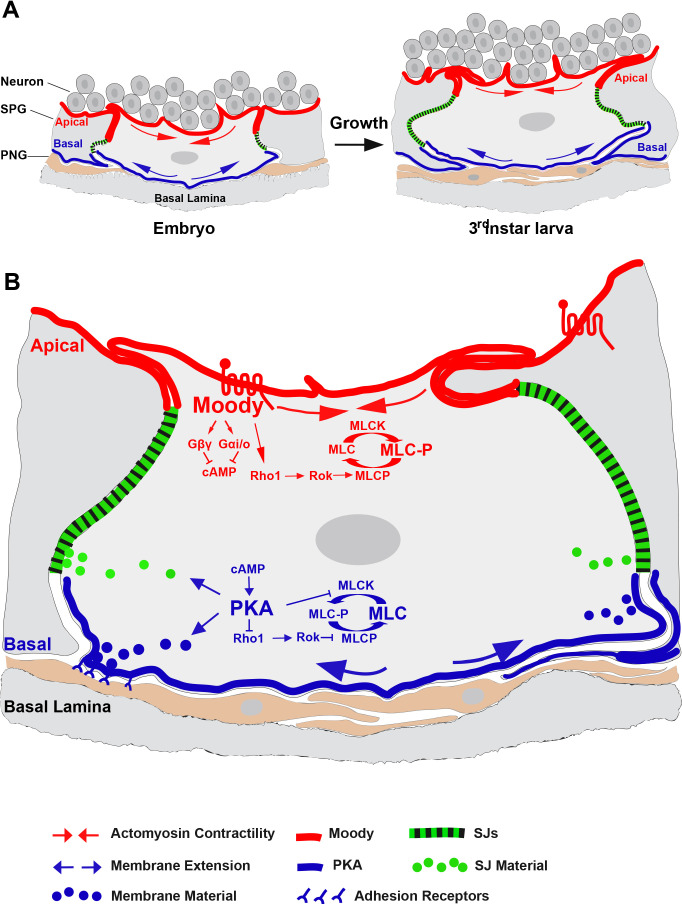
Model of Moody/protein kinase A (PKA) signaling in the glial blood–brain barrier (BBB). Schematic depicting polarized Moody/PKA signaling along the apical–basal axis and its cellular function in controlling subperineural glia (SPG) continued cell growth (**A**) and BBB integrity (**B**) by differentially regulating actomyosin contractility and septate junction (SJ) organization spatiotemporally. For detailed description, see Discussion.

Collectively, our data suggest the following model: polarized Moody/PKA signaling controls the cell growth and maintains BBB integrity during the continuous morphogenesis of the SPG secondary epithelium. On the apical side, Moody activity represses PKA activity (restricting local cAMP level within the apial-basal axis in SPG) and thereby promotes actomyosin contractility. On the basal side, which first adheres to the basal lamina and later to the PNG sheath, PKA activity suppresses actomyosin contractility via MLCK and Rho1 phosphorylation and repression (Figure 7). Throughout development, the SPG grow continuously while extending both their cell surface and expanding their cell contacts. Our data suggest that the membrane extension occurs on the basolateral surface through insertion of plasma membrane and cell-adhesive proteins, with similar behavior in epithelial cell, but regulated by a distinct polarized Moody/PKA signaling in SPG (Wojtal et al., 2008). In analogy to motile cells, the basal side of the SPG would thus act as the ‘leading edge’ of the cell, while the apical side functions as the ‘contractile rear’ (Nelson, 2009). According to this model, Moody/Rho1 regulate actomyosin to generate the contractile forces at the apical side to driving membrane contraction, which directs the basolateral insertion of new membrane material and SJs. In this way, differential contractility and membrane insertion act as a conveyor belt to move new formed membrane contacts and SJ from the basolateral to apical side. Loss of Moody signaling leads to symmetrical localization of PKA and to larger cell contact areas between SPG due to diminished apical constriction. Conversely, loss of PKA causes smaller cell contact areas due to increased basal constriction.

Our results may have important implications for the neuron–glia interaction in the nervous system and the development and maintenance of the BBB in vertebrates. SJs have several structural and functional components in common with paranodal junctions, which join myelinating glial cells to axons in the vertebrate nervous system, and they share similar regulation mechanisms (Hortsch and Margolis, 2003; Salzer, 2003; Salzer, 2015). The vertebrate BBB consists of a secondary epithelium with interdigitations similar to the ones between the *Drosophila* SPG (Chow and Gu, 2015; Cong and Kong, 2020; Hindle and Bainton, 2014; Reinhold and Rittner, 2017). While the sealing is performed by TJs, it will be interesting to investigate whether there are similarities in the underlying molecular and cellular mechanisms that mediate BBB function (Sugimoto et al., 2020).

## Materials and methods

**Key resources table:** 

Reagent type (species) or resource	Designation	Source or reference	Identifiers	Additional information
Genetic reagent (*Drosophila melanogaster*)	*PkaC1^H2^*	Bloomington Drosophila Stock Center	BDSC:4101, RRID:BDSC_4101	FlyBase symbol: Dmel\Pka-C1^H2^
Genetic reagent (*D. melanogaster*)	*PkaC1^B3^*	Bloomington Drosophila Stock Center	FBal0033955	Dmel\Pka-C1^B3^
Genetic reagent (*D. melanogaster*)	*PkaC1^A13^*	Bloomington Drosophila Stock Center	FBal0033953	Dmel\Pka-C1^A13^
Genetic reagent (*D. melanogaster*)	*UAS*mPkaC1*(mC*)	D.Kalderon		DC0
Genetic reagent (*D. melanogaster*)	*moodyGAL4*	T.Schwabe	FBtp0022847	P{moody-GAL4.S}
Genetic reagent (*D. melanogaster*)	*repoGAL4*	Bloomington Drosophila Stock CenterV.Auld	BDSC:7415FBst0007415	P{GAL4}repo
Genetic reagent (*D. melanogaster*)	*Nrg^G305^*	Bloomington Drosophila Stock CenterW.Chia	BDSC:6,844FBal0147727	Dmel\Nrg^G00305^
Genetic reagent (*D. melanogaster*)	*UASGFPMoesin*	D. Kiehart	FBtp0017306	*UAS*mRFPMoesin
Genetic reagent (*D. melanogaster*)	*UAS*mRFPMoesin	T. Schwabe	FBtp0022846	P{UAS-Moe.RFP}
Genetic reagent (*D. melanogaster*)	*Gβ13F^Δ1-96A^*	F. Matsuzaki	FBal0128192	Dmel\Gβ13F^Δ1-96A^
Genetic reagent (*D. melanogaster*)	*UAStauGFP*	M. Krasnow	FBtp0012358	P{UAS-tauGFP}
Genetic reagent (*D. melanogaster*)	*UAS*G_αo_*GTP*	A. Tomlinson	FBal0183487	Dmel\Gαo^GTP.UAS^
Genetic reagent (*D. melanogaster*)	*loco^Δ13^*	C. Klämbt	FBal0096758	Dmel\loco^Δ13^
Genetic reagent (*D. melanogaster*)	*moody^Δ17^*	R. Bainton	FBab0044985	Df(1)moody-Δ17
Genetic reagent (*D. melanogaster*)	*moody-RNAi*	R. Bainton	FBtp0022779	P{UAS-moody.dsRNA}
Genetic reagent (*D. melanogaster*)	*UASnucmCherry*	T. Schwabe		P{UAS-nuc.Cherry}
Genetic reagent (*D. melanogaster*)	*UASGFPEB1*	Bloomington Drosophila Stock CenterD.Brunner	BDSC:35512	Dmel\P{UAS-EB1-GFP}3
Genetic reagent (*D. melanogaster*)	*UASGFPNod*	Bloomington Drosophila Stock Center	FBtp0014112	P{UASp-nod-GFP}
Genetic reagent (*D. melanogaster*)	*UASGFPRho*	Bloomington Drosophila Stock Center	BDSC:9528FBal0189974	Dmel\Rho1^GFP^
Genetic reagent (*D. melanogaster*)	*UASRab4RFP*	Bloomington Drosophila Stock Center	BDSC:8505FBtp0018526	9,562
Genetic reagent (*D. melanogaster*)	*UASactinGFP*	Bloomington Drosophila Stock Center	BDSC:9562FBtp0001557	P{ActGFP}
Genetic reagent (*D. melanogaster*)	*Rho^72R^*	Bloomington Drosophila Stock Center	FBal0061660	Dmel\Rho1^72R^
Genetic reagent (*D. melanogaster*)	*Rho^1B^*	Bloomington Drosophila Stock Center	BDSC:9477FBal0176027	Dmel\Rho1^1B^
Genetic reagent (*D. melanogaster*)	*MLCK^02860^*	Bloomington Drosophila Stock Center	BDSC:11089FBal0159721	Dmel\Strn-Mlck^c02860^
Genetic reagent (*D. melanogaster*)	*MLCK^C234^*	Bloomington Drosophila Stock Center	BDSC:16314FBal0159722	Dmel\Strn-Mlck^C234^
Genetic reagent (*D. melanogaster*)	*tubGAL80^ts^*	Bloomington Drosophila Stock Center	BDSC:7019FBst0007019	Dmel\P{tubP-GAL80^ts^}20
Genetic reagent (*D. melanogaster*)	*PkaC1^KK10896^*	VDRC	VDRC:109758	FBgn0037103
Genetic reagent (*D. melanogaster*)	*Rho1^KK108182^*	VDRC	VDRC:v109420FBal0259627	Dmel\Rho1^KK108182^
Genetic reagent (*D. melanogaster*)	*Tau^GD8682^*	VDRC	VDRC:v25023FBal0210735	Dmel\tau^GD8682^
Antibody	Anti-PkaC1 (rabbit polyclonal)	Lane ME; Genes Dev. 1993	ABS571, RRID:AB_2568479RRID:AB_2568479	IF(1:400)
Antibody	Anti-PkaC1 (mouse monoclonal)	Abcam	ab15051, RRID:AB_2269474	IF(1:500)
Antibody	Anti-REPO (mouse monoclonal)	Developmental Studies Hybridoma Bank	Cat#: 17-9987-42; RRID:AB_2043823	IF(1:10)
Antibody	Anti-GFP (mouse monoclonal)	Molecular Probes	A-11120	IF(1:100)
Antibody	Anti-Mega (mouse monoclonal)	R. Schuh		IF(1:100)
Antibody	Anti-dContactin (guinea pig polyclonal)	M. Bhat		IF(1:100)
Antibody	Anti-RFP (rabbit polyclonal)	US Biological		IF(1:100)
Antibody	Anti-Moody b (rabbit polyclonal)	Bainton et al., 2005; Schwabe et al., 2005		IF(1:500)
Software, algorithm	Fiji	NIH		ImageJ
Software, algorithm	Imaris 4.0	Bitplane		
Chemical compound, drug	Texas red-coupled dextran, 10 kDa	Molecular Probes	D1828	10 mg/ml

### Fly strains and constructs

The following fly strains were obtained from published sources: *PkaC1^H2^* (BDSC Cat# 4101, RRID:BDSC_4101); *PkaC1^B3^; PkaC1^A13^; UAS*mPkaC1*(mC*) (D. Kalderon); *moodyGAL4* (T. Schwabe); *repoGAL4* (V. Auld); *Nrg^G305^* (*NrgGFP*; W.Chia); *UASGFPMoesin* (D.Kiehart); *UAS*mRFPMoesin (T. Schwabe); *Gβ13F^Δ1-96A^* (F. Matsuzaki); *UAStauGFP* (M. Krasnow); *UAS*G_αo_*GTP* (A. Tomlinson), *loco^Δ13^* (C. Klämbt); *moody^Δ17^*(R. Bainton); *moody-RNAi* (R. Bainton); *UASnucmCherry* (T. Schwabe); *UASGFPEB1* (D. Brunner); *UASGFPNod*, *UASGFPRho*, *UASactinGFP*, *UASRab4RFP*, *Rho^72R^*, *Rho^1B^*, *MLCK^02860^*, *MLCK^C234^*, *tubGAL80^ts^* (Bloomington Stock Center); *PkaC1^KK108966^, Rho1^KK108182^, Tau^GD8682^*(VDRC). For live genotyping, mutant and transgenic lines were balanced (*Kr::GFP*) (Casso et al., 1999) or positively marked using *nrgNrgGFP*. Temperature-sensitive control of gene expression in SPG is achieved by using a *tubGAL80ts; moodyGAL4* driver. All strains were raised at 25°C. except for *tubGAL80ts; moodyGAL4* crosses, which were raised at 18°C until 1 day after eclosion and then shifted to 29°.

### Live imaging

Dissected third instar larval cephalic complexes were mounted in PBS and imaged directly. All confocal images were acquired using a Zeiss LSM 510 or 710 system. Stacks of 20–40 0.5-µm confocal sections were generated; image analysis was performed using Zeiss LSM 510, ImageJ (NIH) or Imaris 4.0 (Bitplane) software. The results for each section were assembled as a separate channel of the stack. Time-lapse recordings were carried out on 12 hr *after egg lay (AEL)* embryos raised at 20°C using an inverted Zeiss LSM 510 confocal microscope. To increase signal strength, the pinhole was opened to 1.3 (z-section thickness 0.6 µm), and z-stacks of 12 sections were acquired once per minute. To adjust for focus drift, which is mainly caused by rotation of the embryo, the z-stack coordinates were adjusted at various timepoints without disrupting the continuity of the movie. Between 5 and 7 movies were captured per genotype, each 80–110 min in duration.

### Immunohistochemistry

Immunohistochemistry was performed following standard procedures (Bainton et al., 2005; Schwabe et al., 2005). The antibodies used in the study were rabbit α-PkaC1 (1:400, Pka-C1, RRID:AB_2568479; Lane and Kalderon, 1993), mouse α-PkaC1 (1:100, BD), mouse α-REPO (1:10, Developmental Studies Hybridoma Bank), mouse α-GFP (1:100, Molecular Probes), mouse α-Mega (1:100, R. Schuh), guinea pig α-dContactin (1:1000, M. Bhat), and rabbit α-RFP (1:100, US Biological). Fluorescent secondary antibodies were coupled to Cy3 (1:500, Jackson), Alexa Fluor 488 or Alexa Fluor 633 (1:500, Molecular Probes). Rat α-Moody β was generated in the lab (1:500; Bainton et al., 2005; Schwabe et al., 2005).

### Image analysis

The width of the SJ belt was extracted from maximum intensity projections (MIPs) along the z-axis of 3D confocal stacks of the nervous system. Specifically, we used Imaris 4.0 to perform 2D segmentation of the GFP-marked SJs. For each of the markers, an optimal threshold for the pixel intensity was chosen by fitting the obtained segmented pattern with the raw fluorescence signal. To evaluate the average thickness of the SJs, we splitted the SJ segments into sections of 3–4 µm in length. An approximation of the diameters of the single sections was then obtained by extracting their ellipticity parameters along the axis perpendicular to their main axis. A mean diameter of the SJ was calculated by averaging over the diameters of all single sections. For quantification, random images were chosen with each marker and the distribution of all live markers was measured by Fiji software. To calculate the changes of the distribution of markers, plot profiles of fluorescence intensities along the nucleus to the membrane in each cell were divided into two compartment, membrane area and nucleus area (half to half). Then we calculated the ratio of mean pixel intensity between these two areas (membrane/nucleus) under different PKA activity with each marker. The statistical analysis was performed using Brown–Forsythe and Welch’s ANOVA with multiple comparisons test.

### Dye-penetration assay in embryo, third instar larva, and adult flies

The dye penetration assay in embryos was performed as described (Schwabe et al., 2005). For the dye penetration assay in third instar larvae, a fluorescent dye (Texas red-coupled dextran, 10 kDa, 10 mg/ml, Molecular Probes) was injected into the body cavity of third instar larva. After 2.5 hr, the cephalic complex was dissected, and the dye penetrated into the nerve cord was analyzed using Zeiss LSM710 confocal microscopy. Dye penetration was quantified by calculating the percentage of larva showing dye penetration and by measuring the mean pixel intensity within a representative window of the ventral portion of the nerve cord using Fiji software, and normalized by dividing by the mean of the WT control group. To assess the significance of effects for the embryonic and larval dye penetration assays, Brown–Forsythe and Welch’s ANOVA with multiple comparisons test was performed.

The dye penetration assay in adult flies was performed as described in Bainton et al., 2005 with some critical modifications. Briefly, adult flies were hemolymph injected with 10 mg/ml 10 kDa Texas red-coupled dextran. After 2 hr, the injected flies were decapitated and their heads were mounted in a fluorinated grease-covered glass slides with two compound eyes on the side (the proboscis facing up). Images were acquired on a Zeiss LSM710 confocal microscope at 200–300 μm depths from the eye surface with a Plan Fluor 10xw objective. Dye penetration was quantified by measuring the mean pixel intensities within a representative window of the central region of retina (n = 18–30) of maximum-intensity Z projection of each image stack (z-section thickness 0.6 µm) by Fiji software and normalized by the WT control. Statistical significance was assessed using the two-tailed unpaired t-test.

### Transmission electron microscopy (TEM)

Late stage 17 (22–23 hr AEL) embryos were processed by high-pressure freezing in 20% BSA, freeze-substituted with 2% OsO_4_, 1% glutaraldehyde, and 0.2% uranyl acetate in acetone (90%), dH_2_O (5%), methanol (5%) over 3 days (–90°C to 0°C), washed with acetone on ice, replaced with ethanol, infiltrated and embedded in Spurr’s resin, sectioned at 80 nm and stained with 2% uranyl acetate and 1% lead citrate for 5 min each. Sections were examined with a FEI TECNAI G2 Spirit BioTwin TEM with a Gatan 4K x 4K digital camera. For quantification, random images were shot, and the length of visible SJ membrane stretches in each image was measured using Fiji software. Statistics were calculated using the two-tailed unpaired t-test.

### Serial section transmission electron microscopy (ssTEM)

Freshly dissected third instar larval CNSs were fixed in 2% glutaraldehyde and 2% OsO_4_ in 0.12 M sodium cacodylate (pH 7.4) by microwave (Ted Pella, BioWave Pro MW) as follows: 30" at 300 W, 60" off, 30" at 350 W; 60" off, 30" at 400 W. The samples were then rinsed 2 × 5′ with cold 0.12 M sodium cacodylate buffer; post-fixed with 1% OsO_4_ in 0.12 M sodium cacodylate buffer (pH 7.4) on an ice bath by microwave as follows: 30" at 350 W, 60" off, 30" at 375 W, 60" off, 30" at 400 W; rinsed 2 × 5′ with 0.12 M sodium cacodylate buffer at RT; 2 × 5′ with distilled water at RT; stained in 1% uranyl acetate overnight in 4°C; rinsed 6 × 5′ with distilled water; dehydrated with ethanol followed by propylene oxide (15′); infiltrated and embedded in Eponate 12 with 48 hr polymerization in a 65°C oven. 50 nm serial sections were cut on a Leica UC6 ultramicrotome and picked up with Synaptek slot grids on a carbon-coated Pioloform film. Sections were post-stained with 1% uranyl acetate followed by Sato’s (1968) lead. The image acquisition of multiple sections (~150 sections in each genotype) and large tissue areas was automatically captured with a Gatan 895 4K × 4K camera by a FEI Spirit TECNAI BioTWIN TEM using Leginon (Suloway et al., 2005). TrakEM2 software was used to montage, align images, trace, and reconstruct 3D SJ structures between contacting SPG within and across serial sections. For quantification, random images were chosen, and the length of visible SJs stretches and membrane contacting area in each image was measured using Fiji. The statistical analysis was performed using Brown–Forsythe and Welch’s ANOVA with multiple comparisons test.

## Data Availability

All data generated or analysed during this study are included in Dyrad generic databases with DOI https://doi.org/10.5061/dryad.fj6q573tx. Source data files have been provided for Figures 1, 2, 3, 4, 6 and Figure supplement 2 and 5. The following dataset was generated: LiX FetterR SchwabeT JungC LiuL StellerH GaulU 2021The cAMP effector PKA mediates Moody GPCR signaling in *Drosophila* blood-brain barrier formation and maturationDryad Digital Repository10.5061/dryad.fj6q573txPMC839000334382936

## Additional files

Transparent reporting form

## References

[bib1] Artiushin G, Zhang SL, Tricoire H, Sehgal A (2018). Endocytosis at the *Drosophila* blood-brain barrier as a function for sleep. eLife.

[bib2] Babatz F, Naffin E, Klämbt C (2018). The *Drosophila* blood-brain barrier adapts to cell growth by unfolding of pre-existing septate junctions. Developmental Cell.

[bib3] Bainton RJ, Tsai LT-Y, Schwabe T, DeSalvo M, Gaul U, Heberlein U (2005). moody encodes two GPCRs that regulate cocaine behaviors and blood-brain barrier permeability in *Drosophila*. Cell.

[bib4] Banerjee S, Sousa AD, Bhat MA (2006). Organization and function of septate junctions: An evolutionary perspective. Cell Biochemistry and Biophysics.

[bib5] Bauman AL, Goehring AS, Scott JD (2004). Orchestration of synaptic plasticity through AKAP signaling complexes. Neuropharmacology.

[bib6] Behr M, Riedel D, Schuh R (2003). The claudin-like megatrachea is essential in septate junctions for the epithelial barrier function in *Drosophila*. Developmental Cell.

[bib7] Cardona A, Saalfeld S, Schindelin J, Arganda-Carreras I, Preibisch S, Longair M, Tomancak P, Hartenstein V, Douglas RJ (2012). TRAKEM2 software for neural circuit reconstruction. PLOS ONE.

[bib8] Carlson SD, Juang JL, Hilgers SL, Garment MB (2000). Blood barriers of the insect. Annual Review of Entomology.

[bib9] Casso D, Ramirez-Weber FA, Kornberg TB (1999). GFP-tagged balancer chromosomes for *Drosophila melanogaster*. Mechanisms of Development.

[bib10] Chen X, Ganetzky B (2012). A neuropeptide signaling pathway regulates synaptic growth in *Drosophila*. The Journal of Cell Biology.

[bib11] Chow BW, Gu C (2015). The molecular constituents of the blood-brain barrier. Trends in Neurosciences.

[bib12] Clark IE, Jan LY, Jan YN (1997). Reciprocal localization of Nod and kinesin fusion proteins indicates microtubule polarity in the *Drosophila* oocyte, epithelium, neuron and muscle. Development.

[bib13] Cong X, Kong W (2020). Endothelial tight junctions and their regulatory signaling pathways in vascular homeostasis and disease. Cellular Signalling.

[bib14] Cui W, Sproul LR, Gustafson SM, Matthies HJG, Gilbert SP, Hawley RS (2005). *Drosophila* nod protein binds preferentially to the plus ends of microtubules and promotes microtubule polymerization in vitro. Molecular Biology of the Cell.

[bib15] Deligiannaki M, Casper AL, Jung C, Gaul U (2015). Pasiflora proteins are novel core components of the septate junction. Development.

[bib16] Dong JM, Leung T, Manser E, Lim L (1998). cAMP-induced Morphological Changes Are Counteracted by the Activated RhoA Small GTPase and the Rho Kinase ROKα. Journal of Biological Chemistry.

[bib17] Edwards JS, Swales LS, Bate M (1993). The differentiation between neuroglia and connective tissue sheath in insect ganglia revisited: the neural lamella and perineurial sheath cells are absent in a mesodermless mutant of *Drosophila*. The Journal of Comparative Neurology.

[bib18] Faivre-Sarrailh C, Banerjee S, Li JJ, Hortsch M, Laval M, Bhat MA (2004). *Drosophila* contactin, a homolog of vertebrate contactin, is required for septate junction organization and paracellular barrier function. Development.

[bib19] Fessler LI, Nelson RE, Fessler JH (1994). *Drosophila* extracellular matrix. Methods in Enzymology.

[bib20] Garcia JG, Davis HW, Patterson CE (1995). Regulation of endothelial cell gap formation and barrier dysfunction: role of myosin light chain phosphorylation. Journal of Cellular Physiology.

[bib21] Garcia JG, Lazar V, Gilbert-McClain LI, Gallagher PJ, Verin AD (1997). Myosin light chain kinase in endothelium: Molecular cloning and regulation. American Journal of Respiratory Cell and Molecular Biology.

[bib22] Garcia JGN, Verin AD, Schaphorst K, Siddiqui R, Patterson CE, Csortos C, Natarajan V (1999). Regulation of endothelial cell myosin light chain kinase by rho, cortactin, and p60 SRC. American Journal of Physiology-Lung Cellular and Molecular Physiology.

[bib23] Genova JL, Fehon RG (2003). Neuroglian, gliotactin, and the na+/k+ atpase are essential for septate junction function in *Drosophila*. The Journal of Cell Biology.

[bib24] Granderath S, Stollewerk A, Greig S, Goodman CS, O’Kane CJ, Klämbt C (1999). LOCO encodes an RGS protein required for *Drosophila* glial differentiation. Development.

[bib25] Guan Z, Buhl LK, Quinn WG, Littleton JT (2011). Altered gene regulation and synaptic morphology in *Drosophila* learning and memory mutants. Learning & Memory.

[bib26] Halter DA, Urban J, Rickert C, Ner SS, Ito K, Travers AA, Technau GM (1995). The homeobox gene repo is required for the differentiation and maintenance of glia function in the embryonic nervous system of *Drosophila melanogaster*. Development.

[bib27] Hartenstein V (2011). Morphological diversity and development of glia in *Drosophila*. Glia.

[bib28] Hijazi A, Masson W, Augé B, Waltzer L, Haenlin M, Roch F (2009). boudin is required for septate junction organisation in *Drosophila* and codes for a diffusible protein of the Ly6 superfamily. Development.

[bib29] Hijazi A, Haenlin M, Waltzer L, Roch F (2011). The ly6 protein coiled is required for septate junction and blood brain barrier organisation in *Drosophila*. PLOS ONE.

[bib30] Hindle SJ, Bainton RJ (2014). Barrier mechanisms in the *Drosophila* blood-brain barrier. Frontiers in Neuroscience.

[bib31] Hortsch M, Margolis B (2003). Septate and paranodal junctions: Kissing cousins. Trends in Cell Biology.

[bib32] Howe AK (2004). Regulation of actin-based cell migration by CAMP/PKA. Biochimica et Biophysica Acta.

[bib33] Ito K, Urban J, Technau GM (1995). Distribution, classification, and development of *Drosophila* glial cells in the late embryonic and early larval ventral nerve cord. Roux’s Archives of Developmental Biology.

[bib34] Izumi Y, Furuse M (2014). Molecular organization and function of invertebrate occluding junctions. Seminars in Cell & Developmental Biology.

[bib35] Jarecki J, Johnson E, Krasnow MA (1999). Oxygen regulation of airway branching in *Drosophila* is mediated by branchless FGF. Cell.

[bib36] Kalderon D, Rubin GM (1988). Isolation and characterization of *Drosophila* camp-dependent protein kinase genes. Genes & Development.

[bib37] Lane ME, Kalderon D (1993). Genetic investigation of camp-dependent protein kinase function in *Drosophila* development. Genes & Development.

[bib38] Lane ME, Kalderon D (1994). RNA localization along the anteroposterior axis of the *Drosophila* oocyte requires pka-mediated signal transduction to direct normal microtubule organization. Genes & Development.

[bib39] Lane ME, Kalderon D (1995). Localization and functions of Protein kinase A during *Drosophila oogenesis*. Mechanisms of Development.

[bib40] Lang P, Gesbert F, Delespine-Carmagnat M, Stancou R, Pouchelet M, Bertoglio J (1996). Protein kinase a phosphorylation of Rhoa mediates the morphological and functional effects of cyclic AMP in cytotoxic lymphocytes. The EMBO Journal.

[bib41] Li W, Ohlmeyer JT, Lane ME, Kalderon D (1995). Function of protein kinase A in hedgehog signal transduction and *Drosophila* imaginal disc development. Cell.

[bib42] Li W, Tully T, Kalderon D (1996). Effects of a conditional *Drosophila* PKA mutant on olfactory learning and memory. Learning & Memory.

[bib43] Li D, Liu Y, Pei C, Zhang P, Pan L, Xiao J, Meng S, Yuan Z, Bi X (2017). Mir-285-yki/mask double-negative feedback loop mediates blood-brain barrier integrity in *Drosophila*. PNAS.

[bib44] Lim CJ, Kain KH, Tkachenko E, Goldfinger LE, Gutierrez E, Allen MD, Groisman A, Zhang J, Ginsberg MH (2008). Integrin-mediated Protein kinase a activation at the leading edge of migrating cells. Molecular Biology of the Cell.

[bib45] Llimargas M, Strigini M, Katidou M, Karagogeos D, Casanova J (2004). Lachesin is a component of a septate junction-based mechanism that controls tube size and epithelial integrity in the *Drosophila* tracheal system. Development.

[bib46] Machacek M, Hodgson L, Welch C, Elliott H, Pertz O, Nalbant P, Abell A, Johnson GL, Hahn KM, Danuser G (2009). Coordination of RHO gtpase activities during cell protrusion. Nature.

[bib47] Magie CR, Parkhurst SM (2005). Rho1 regulates signaling events required for proper *Drosophila* embryonic development. Developmental Biology.

[bib48] Marks SA, Kalderon D (2011). Regulation of mammalian Gli proteins by Costal 2 and PKA in *Drosophila* reveals Hedgehog pathway conservation. Development.

[bib49] Mayer F, Mayer N, Chinn L, Pinsonneault RL, Kroetz D, Bainton RJ (2009). Evolutionary conservation of vertebrate blood-brain barrier chemoprotective mechanisms in *Drosophila*. The Journal of Neuroscience.

[bib50] Nelson WJ (2009). Remodeling epithelial cell organization: Transitions between front-rear and apical-basal polarity. Cold Spring Harbor Perspectives in Biology.

[bib51] Olofsson B, Page DT (2005). Condensation of the central nervous system in embryonic *Drosophila* is inhibited by blocking hemocyte migration or neural activity. Developmental Biology.

[bib52] Oshima K, Fehon RG (2011). Analysis of protein dynamics within the septate junction reveals a highly stable core protein complex that does not include the basolateral polarity protein discs large. Journal of Cell Science.

[bib53] Park SK, Sedore SA, Cronmiller C, Hirsh J (2000). Type II camp-dependent protein kinase-deficient *Drosophila* are viable but show developmental, circadian, and drug response phenotypes. The Journal of Biological Chemistry.

[bib54] Petri J, Syed MH, Rey S, Klämbt C (2019). Non-cell-autonomous function of the gpi-anchored protein UNDICHT during septate junction assembly. Cell Reports.

[bib55] Reinhold AK, Rittner HL (2017). Barrier function in the peripheral and central nervous system-a review. Pflugers Archiv.

[bib56] Renger JJ, Ueda A, Atwood HL, Govind CK, Wu CF (2000). Role of cAMP cascade in synaptic stability and plasticity: ultrastructural and physiological analyses of individual synaptic boutons in *Drosophila* memory mutants. The Journal of Neuroscience.

[bib57] Rogers SL, Wiedemann U, Hacker U, Turck C, Vale RD (2004). *Drosophila* RhoGEF2 associates with microtubule plus ends in an EB1-dependent manner. Current Biology.

[bib58] Salzer JL (2003). Polarized domains of myelinated axons. Neuron.

[bib59] Salzer JL, Brophy PJ, Peles E (2008). Molecular domains of myelinated axons in the peripheral nervous system. Glia.

[bib60] Salzer JL (2015). Schwann cell myelination. Cold Spring Harbor Perspectives in Biology.

[bib61] Schindelin J, Arganda-Carreras I, Frise E, Kaynig V, Longair M, Pietzsch T, Preibisch S, Rueden C, Saalfeld S, Schmid B, Tinevez J-Y, White DJ, Hartenstein V, Eliceiri K, Tomancak P, Cardona A (2012). Fiji: An open-source platform for biological-image analysis. Nature Methods.

[bib62] Schwabe T, Bainton RJ, Fetter RD, Heberlein U, Gaul U (2005). GPCR signaling is required for blood-brain barrier formation in *Drosophila*. Cell.

[bib63] Schwabe T, Li X, Gaul U (2017). Dynamic analysis of the mesenchymal-epithelial transition of blood-brain barrier forming glia in *Drosophila*. Biology Open.

[bib64] Shabb JB (2001). Physiological substrates of camp-dependent protein kinase. Chemical Reviews.

[bib65] Stork T, Engelen D, Krudewig A, Silies M, Bainton RJ, Klambt C (2008). Organization and function of the blood-brain barrier in *Drosophila*. The Journal of Neuroscience.

[bib66] Sugimoto K, Ichikawa-Tomikawa N, Nishiura K, Kunii Y, Sano Y, Shimizu F, Kakita A, Kanda T, Imura T, Chiba H (2020). Serotonin/5-ht1a signaling in the neurovascular unit regulates endothelial cldn5 expression. International Journal of Molecular Sciences.

[bib67] Suloway C, Pulokas J, Fellmann D, Cheng A, Guerra F, Quispe J, Stagg S, Potter CS, Carragher B (2005). Automated molecular microscopy: The new leginon system. Journal of Structural Biology.

[bib68] Tang S -t, Tang H -q, Su H, Wang Y, Zhou Q, Zhang Q, Wang Y, Zhu H -q (2019). Glucagon-like peptide-1 attenuates endothelial barrier injury in diabetes via CAMP/PKA mediated down-regulation of MLC phosphorylation. Biomedicine & Pharmacotherapy.

[bib69] Taylor SS, Buechler JA, Yonemoto W (1990). Camp-dependent protein kinase: Framework for a diverse family of regulatory enzymes. Annual Review of Biochemistry.

[bib70] Tempesta C, Hijazi A, Moussian B, Roch F (2017). Boudin trafficking reveals the dynamic internalisation of specific septate junction components in *Drosophila*. PLOS ONE.

[bib71] Tepass U, Hartenstein V (1994). The development of cellular junctions in the *Drosophila* embryo. Developmental Biology.

[bib72] Tepass U, Tanentzapf G, Ward R, Fehon R (2001). Epithelial cell polarity and cell junctions in *Drosophila*. Annual Review of Genetics.

[bib73] Tkachenko E, Sabouri-Ghomi M, Pertz O, Kim C, Gutierrez E, Machacek M, Groisman A, Danuser G, Ginsberg MH (2011). Protein kinase a governs a RHOA-RHOGDI protrusion-retraction pacemaker in migrating cells. Nature Cell Biology.

[bib74] Unhavaithaya Y, Orr-Weaver TL (2012). Polyploidization of Glia in neural development links tissue growth to blood-brain barrier integrity. Genes & Development.

[bib75] Vasin A, Zueva L, Torrez C, Volfson D, Littleton JT, Bykhovskaia M (2014). Synapsin regulates activity-dependent outgrowth of synaptic boutons at the *Drosophila* neuromuscular junction. The Journal of Neuroscience.

[bib76] Verin AD, Gilbert-McClain LI, Patterson CE, Garcia JGN (1998). Biochemical regulation of the nonmuscle myosin light chain kinase isoform in bovine endothelium. American Journal of Respiratory Cell and Molecular Biology.

[bib77] Von Stetina JR, Frawley LE, Unhavaithaya Y, Orr-Weaver TL (2018). Variant cell cycles regulated by notch signaling control cell size and ensure a functional blood-brain barrier. Development.

[bib78] Wojtal KA, Hoekstra D, van Ijzendoorn SCD (2008). Camp-dependent Protein kinase A and the dynamics of epithelial cell surface domains: Moving membranes to keep in shape. BioEssays.

[bib79] Wu VM, Schulte J, Hirschi A, Tepass U, Beitel GJ (2004). Sinuous is a *Drosophila* claudin required for septate junction organization and epithelial tube size control. The Journal of Cell Biology.

[bib80] Xu N, Myat MM (2012). Coordinated control of lumen size and collective migration in the salivary gland. Fly.

[bib81] Yi P, Johnson AN, Han Z, Wu J, Olson EN (2008). Heterotrimeric G proteins regulate a noncanonical function of septate junction proteins to maintain cardiac integrity in *Drosophila*. Developmental Cell.

[bib82] Zhang J, Schulze KL, Hiesinger PR, Suyama K, Wang S, Fish M, Acar M, Hoskins RA, Bellen HJ, Scott MP (2007). Thirty-one flavors of *Drosophila* rab proteins. Genetics.

[bib83] Zhou Q, Apionishev S, Kalderon D (2006). The contributions of protein kinase A and smoothened phosphorylation to hedgehog signal transduction in *Drosophila melanogaster*. Genetics.

